# *Alishewanella* Phage LSH1 from the Sea Surface Microlayer Provides a Novel Minimalistic View of the Siphoviral Hub Structure

**DOI:** 10.34133/csbj.0131

**Published:** 2026-06-08

**Authors:** Stephen C. Hardies, Jihye Park, Byung Cheol Cho, Chung Yeon Hwang

**Affiliations:** ^1^ Department of Biochemistry and Structural Biology, UT Health, San Antonio, TX, USA.; ^2^ Microbial Oceanography Laboratory, School of Earth and Environmental Sciences and Research Institute of Oceanography, Seoul National University, Seoul, Republic of Korea.

## Abstract

LSH1 is a novel lytic siphovirus isolated, together with its host in the genus *Alishewanella*, from the surface microlayer of a brackish tidal reservoir in South Korea and characterized with respect to growth properties, genome sequence, gene annotation, mass spectrometry, and electron microscopy. Sequence analysis shows that LSH1 shares only distant similarity to other cultured phages, although a closer metagenomic neighborhood can be defined. LSH1 represents the first isolate from a large, previously unsampled family-level sector of the viral tree. Transmission electron microscopy revealed a tail end distinct from the best structurally characterized siphoviral prototypes and similar in appearance to *Salmonella* phage Jersey, the prototype of a large structurally uncharacterized group named *Guernseyvirinae.* Therefore, Jersey was included in the comparative analysis with LSH1. A combination of hidden Markov model comparisons and AlphaFold reconstruction was used to clarify the structural relationships of these phages. Both have structural homologs of portions of the canonical bacteriophage lambda tail hub but lack the lambda components associated with receptor recognition linked to ejection triggering in that system. The LSH1 and Jersey tail hubs are of different sequence lineages, but each represents a relatively minimalistic version of the siphoviral tail hub, with distinct candidates for the structural location of their antireceptors. This study explores the capability of AlphaFold to rapidly augment the relatively few structurally characterized phages with models for diverse variants, fleshing out how much variation there is and perhaps leading to a better treatment of how this variation is evolving.

## Introduction

We have been isolating bacteria and their phages from unusual oceanic sites in an effort to more fully understand the microbiology of these environments. The sea surface microlayer (SML), defined as the upper tens to hundreds of micrometers of the ocean’s surface, forms a biogeochemically distinctive habitat at the air–sea interface [1]. This layer is enriched in surfactants, transparent exopolymeric particles, and polysaccharide gels, creating a viscous and gelatinous environment [2]. Such organic enrichment promotes the rapid growth of copiotrophic heterotrophic bacteria, including *Vibrio* and *Pseudoalteromonas*, which thrive under high substrate availability and play vital roles in coastal carbon cycling [3]. In coastal SMLs, bacterial and viral abundances are often higher than in subsurface waters, suggesting increased probabilities of phage–host encounters [4,5]. Under these conditions, characterized by active bacterial growth and elevated encounter rates, the SML provides an ideal natural microcosm for investigating phage–host dynamics and coevolution. Recent studies have identified distinct viral–bacterial communities and novel phage–host systems in the SML [6,7]. These findings highlight the coastal SML as a critical yet underexplored environment for isolating and characterizing phage–host systems, advancing our understanding of microbial ecology and evolutionary processes at the ocean’s surface.

A novel *Alishewanella* host–phage system including phage LSH1 was isolated from a coastal SML. One objective of this study is to characterize the general growth properties of LSH1. Another objective is to produce a higher-than-average level of annotation in the sequence database entry. This mainly employs the HHpred system to generate family database references ideally leading to a literature citation, and to sort out whether there are multiple functionally distinct domains in an individual polypeptide. Our paper introduces the GenBank entry, but mostly focuses on a third objective, which is to use AlphaFold 3 multimer modeling capability to augment the understanding of some virion structural proteins for which questions persist after annotating up to the level of HHpred. We were encouraged to explore this route because AlphaFold 3 has shown it can make protein structural predictions from sequence with excellent performance in a Critical Assessment of Protein Structure Prediction competition [8].

This paper focuses on virion structure proteins because we have performed mass spectrometry to know which proteins those are, and transmission electron microscopy to know that LSH1 is a siphovirus (a tailed phage with a long noncontractile tail). There is substantial prior information available from comparative studies of cryo-electron microscopy (cryoEM) structures of different siphoviruses [9,10]. The gram-negative prototypes most often mentioned are *Escherichia* phages lambda [11,12] and T5 [13]. Since HHpred relates the LSH1 proteins in question most strongly to these, we will focus on comparison to them. See the reviews above for others that illustrate the global consistency and variability of the siphoviral tail end, including *Bacillus* phage SPP1 [14] and the related *Lactococcus* phage J-1 [15] and other phages of gram-positive hosts. Moreover, there are additional cryoEM structures appearing at an increasing rate.

The structural proteins of LSH1 for which HHpred had left questions include the tail tube, the distal tail protein, the tail hub, a putative side fiber, and a 3-protein novel structural module. Our analysis is focused on domain-sized alterations envisioned in an AlphaFold multiconstruct of a structural subassembly. All of the AlphaFold work is done through the publicly available Deep Mind server. These 5 targets offer different bioinformatic challenges.

The tail tube and distal tail protein have an extremely recognizable fold and consistent geometry across all long-tailed phages, but frequently carry a decoration domain that differs among phages. After HHpred analysis, there seemed to be novel decorations in each of these proteins (at least as far as HHpred could tell). An objective for both proteins was to try to relate those decorations to others already reported in the literature. An additional objective for the distal tail protein was to use it to anchor the hub subassembly construction, based on the knowledge in the citations given above that the hub/distal tail protein interface is conserved.

From the comparative studies cited above, it is clear that the hub protein forms a complex together with a distal tail protein at the tail end of siphoviruses with consistent 3-fold stoichiometry and a recognizable core geometry. A hub subunit can be encoded by one polypeptide as in T5 and most other phages, or by as many as 3 polypeptides as in lambda. It is subjected to variation by both translational fusion and noncovalent association with a dizzying variety of auxiliary tail proteins. Hence, the hub is expected to be a target with extensive prior information but with lots of domain-sized variation to sort out.

Appendages emanating from the side of the phage tail end are called side fibers here to distinguish from central fibers that extend as a single appendage from the tail end. Phage appendages are often referred to as “tail spikes”, which sometimes just means a fiber, but other times means a particular structural morphology, so we prefer not to use it. Side fibers are notoriously mosaic and may be composed of one or several polypeptides [16,17], and are often excluded from cryoEM models, presumably because they are conformationally flexible. Classic side fibers are trimeric [18,19], attaching to the virion by an N-terminal domain and ending in a C-terminal domain that may function either in adhesion or depolymerization of interfering biofilm polymers. There may be segments of the side fiber encoded in different polypeptides. There is a growing array of appendages that do not have the classic trimeric structure, and there may be some that have the opposite polarity. Hence, the objective with the LSH1 side fiber is to clarify if it is of the classic trimeric form, discover if there is a mosaic pattern of matches to other characterized phage appendages, and, if possible, determine how it attaches to the virion.

The novel 3-protein module is an exercise at starting with virtually no prior information except that it must attach to the virion somehow or else it would not be in the mass spectrometry survey. Hence, we are interested in anything that can be learned about it.

As part of the preliminary characterization of LSH1, we will describe how it falls within the developing official phage taxonomy managed by the International Committee on the Taxonomy of Viruses (ICTV). ICTV has removed morphology from its taxonomy system [20]. The official taxonomic names are given either in the treatment of *Guernseyvirinae* given below or in Materials and Methods. Since the relevant character of the protein structures discussed here is to determine morphology, we will refer to the various phages discussed as podoviruses (short tail), siphoviruses (long noncontractile tail), or myoviruses (long contractile tail), to keep those morphological distinctions in sight. While working on the taxonomic problem, we encountered another taxon now named *Guernseyvirinae* based on the prototype *Salmonella* phage Jersey, which had some similarity in the somewhat minimal appearance of the LSH1 tail end as seen by electron microscopy (EM). The Jersey was included in the study with the objective to see if it was more closely related to LSH1 at the molecular level or coincidentally just had a less complex tail end.

## Results

### Isolation and growth properties

Two environmental bacterial strains and phage LSH1 were isolated from SML water (thickness ~40 μm) at the coast of South Korea, as described in Materials and Methods. The strains HL-SH05 and HL-SH06 were identified as members of the genus *Alishewanella* based on whole-genome sequencing and phylogenomic analysis of 120 concatenated marker genes (Fig. S1). Phage LSH1 was isolated using HL-SH05 as the host and formed plaques on this strain, but not on HL-SH06 or closely related *Alishewanella maricola* KACC 22238^T^ (average nucleotide identity [ANI] 98.3% and 97.9%, respectively), indicating a narrow host range. The phage formed clear plaques and showed no indication of lysogeny. Other measured growth properties of LSH1 included an adsorption constant of 6.7 × 10^−9^, a latent period of 120 min, and a burst size of 68 ± 7 (mean ± SD) at 30 °C (Fig. S2). The phage was resistant to 10% chloroform at 30 °C for an hour and exhibited maximum stability at pH 7. Thermal stability assays showed that infectivity was maintained between –20 and 40 °C, partially reduced at 50 and 60 °C, and completely lost at 70 °C.

### Electron microscopy

**Fig. 1.**
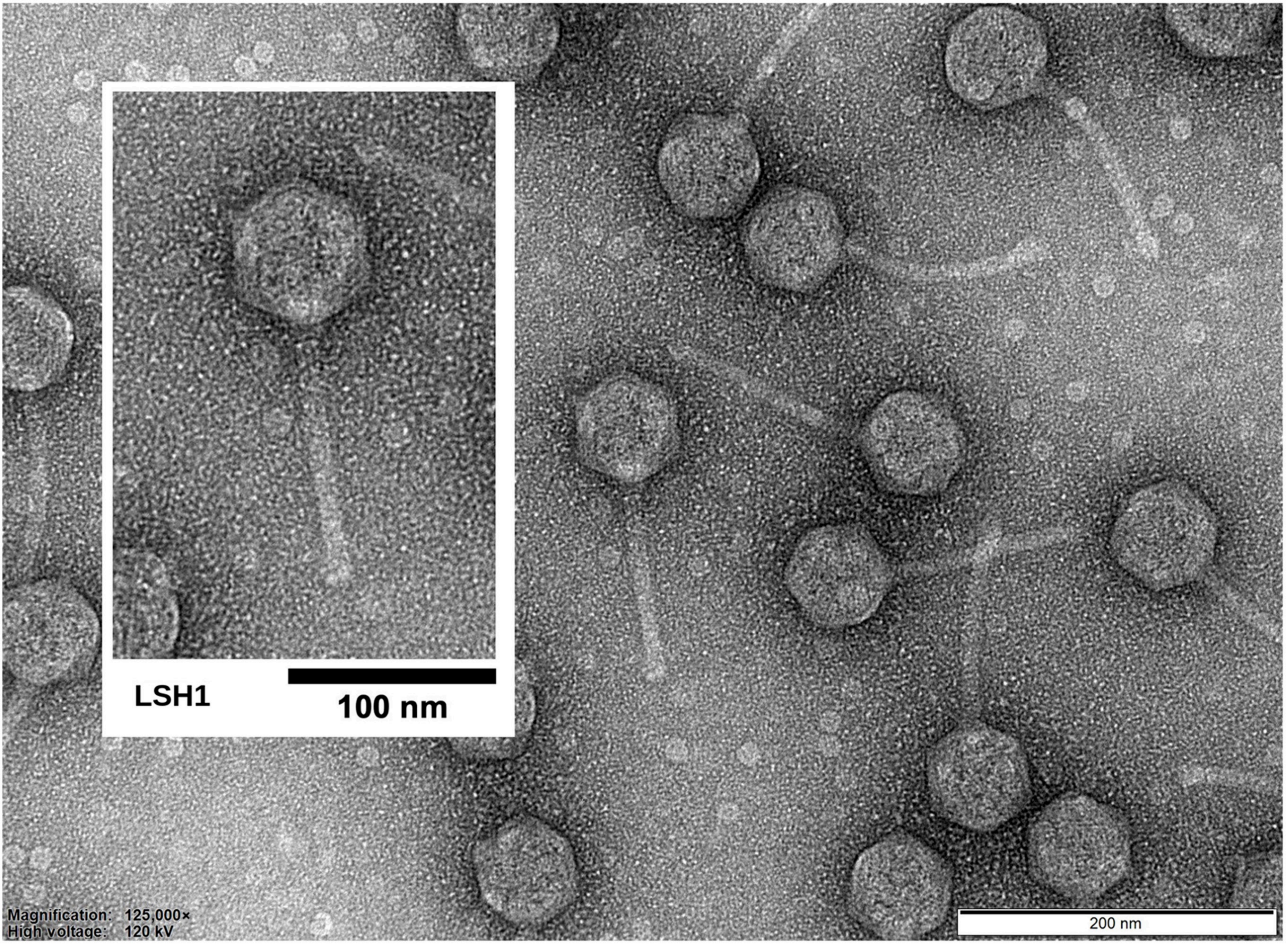
Transmission electron microscopy of *Alishwanella* phage LSH1.

EM of LSH1 showed a long tail typical of siphoviruses (Fig. 1). The tail end appears nonconical and squared off, unlike lambda [11,12] or T5 [13] tail ends. LSH1 may have some protruding elements at the tail end, although these are difficult to see or count. EM images from *Guernseyvirinae* show more prominent protrusions, e.g., Refs. [21,22]. LSH1 gp26 has limited sequence similarity near the N-terminus of Jersey gp33, leaving the question of whether it is attached in the same way, and why we do not see a side fiber more clearly in the LSH1 EM.

### Prior information on *Salmonella* phage Jersey morphology

The EM appearance of LSH1 seemed similar to the phage *Salmonella* phage Jersey. Ackermann [23] initially designated Jersey as a novel siphoviral morphotype because of the appearance of the tail end appendages. He drew these in diagrammatic form as lines extending from a squared off tail end laterally and downwards, each terminating in a small sphere, and estimated that there were 6 of them per virion. Sequence similarity of Jersey gp33 to the P22 6-fold tail spike has been noted [21], and indeed, the EM appearance of lateral protrusions from P22 and the Jersey tail ends is similar, e.g., Ref. [24]. The P22 tail spike is attached in 6-fold symmetry to the P22 podoviral tail nozzle [18], which has no homolog in the siphoviral structure.

### Sequence annotation

**Fig. 2.**
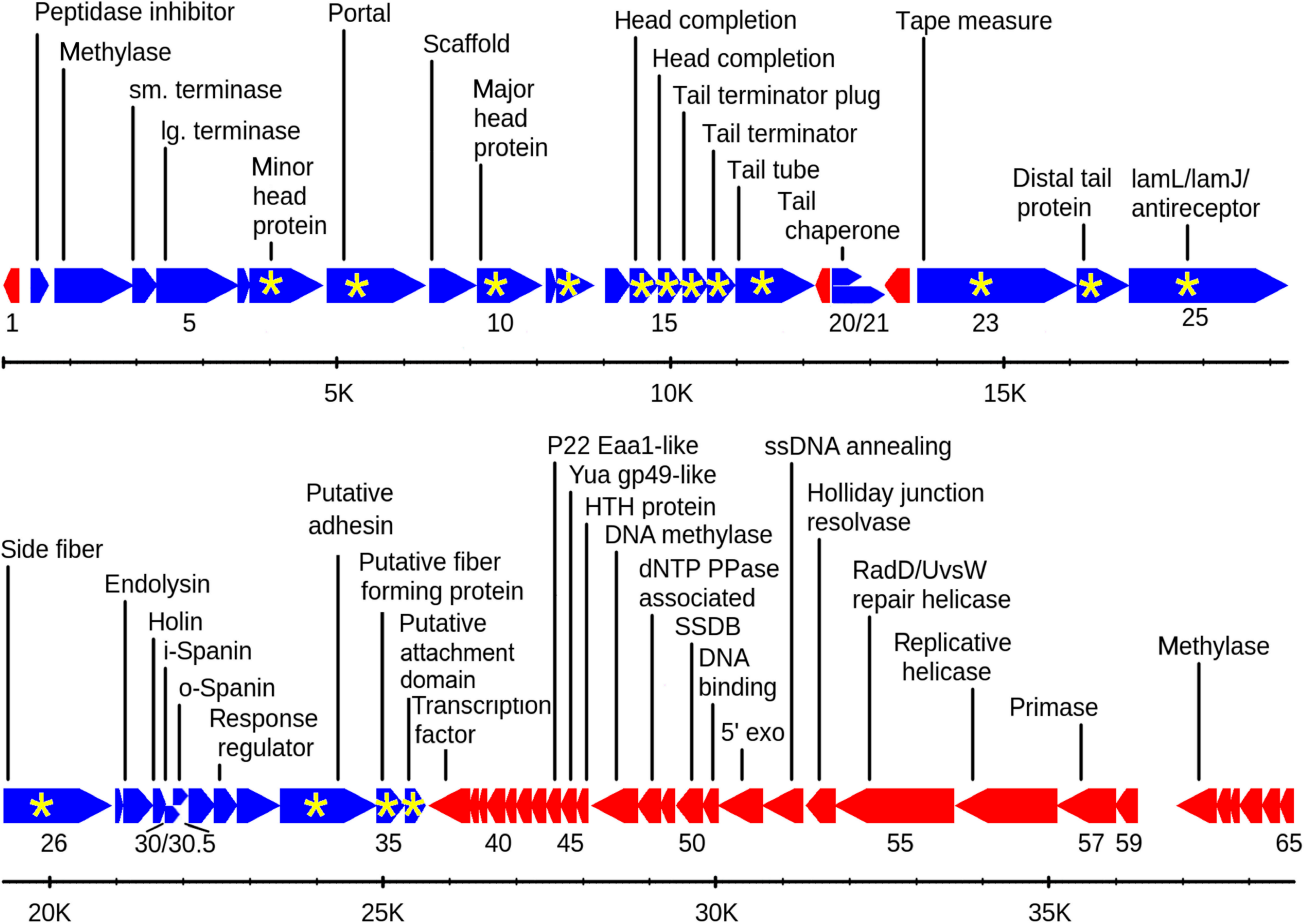
Gene organization of LSH1. Blue and red indicate forward and reverse strand genes. Asterisks indicate a virion protein detected by mass spectrometry. The lamL annotation is from the use of AlphaFold described here.

LSH1 reading frames were annotated with the HHpred hidden Markov model (HMM)–HMM comparison system. Mass spectrometry was performed as described in Materials and Methods to identify virion proteins (Fig. 2 and Table S1). HHpred matches clarifying the nature of the encoded proteins and other clarifying information are given in GenBank accession PX020959. Figure 2 shows a map labeling the genes for which some functional inference is available. LSH1 has a typical organization of phage genomes of this size, featuring 2 opposing major transcription modules with one (rightwards) devoted to structure, morphogenesis, and lysis, and the other (leftwards) devoted to early functions, replication, recombination, and repair. There is a cluster of 3 encoded virion proteins at the end of the rightwards unit that do not conform to the usual set of siphoviral virion proteins (gp34 to 36). Clusters of small nondescript genes appear in the leftwards transcription unit (encoding gp37 to 46 and gp61 to 65), presumably including host takeover functions. Proteins in the head and head/tail connector modules (gp7 to 17) generally matched better to SPP1 HMM models than to lambda HMM models; the tail tube (gp18) matched better to the T5 tail tube HMM; and the tail end proteins matched, although somewhat less convincingly to lambda HMM models. Gp24 matched weakly (*E* = 0.021), and gp25 matched well to a portion of the lambda hub protein gpJ (*E* = 10^−10^), but not at all to the lambda hub proteins gpL and gpI. These observations established that there is mosaicism in the evolution of the LSH1 structural modules, as is nearly always the case, with different ancestry of the head and tail modules, and that the tail end had a particular need for further clarification.

### Phage classification

**Fig. 3.**
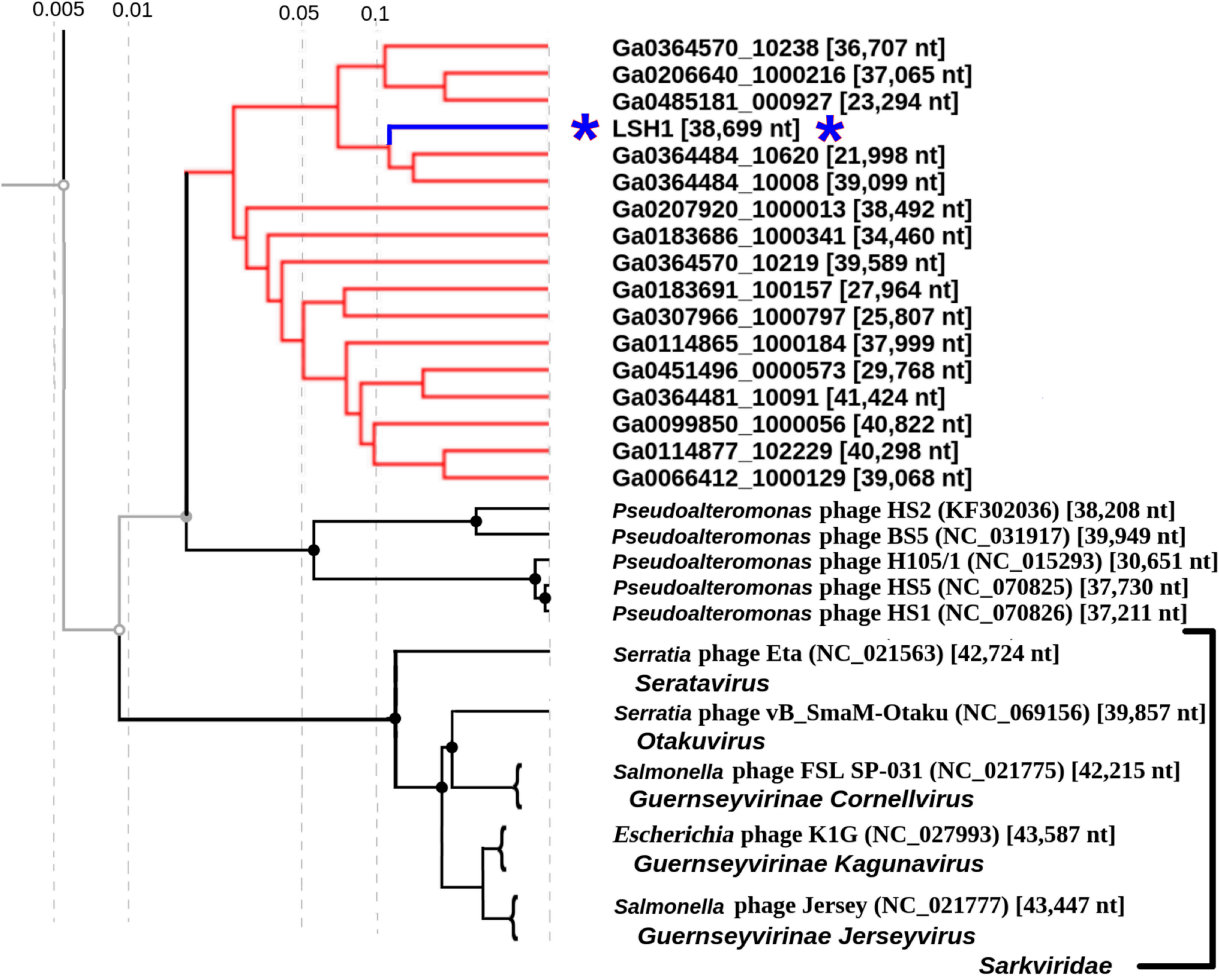
ViPTree analysis of LSH1 relative to weakly related *Pseudoalteromonas* phages and representatives of the family *Sarkviridae*. Added IMGVR metagenomic sequences are in red. Braces indicate a large clade collapsed around one representative.

LSH1 has no nucleotide similarity to any phage in GenBank. It does have blastP matches and Psi-Blast matches to other phages. A tool to ask if the protein matches might join LSH1 to any established ICTV taxon is the ViPTree server (Fig. 3). LSH1 does not join closely with any named phages, although a neighborhood around LSH1 can be filled in with metagenomic phage genomes from the virus division of the Integrated Microbial Genomes/Virus Resource (IMGVR) database [25] (Table S2 and Materials and Methods). Because the process of horizontal transfer and recombination [26] introduces increasing numbers of genes with conflicting ancestry into phage genomes with increasing divergence time, ICTV, citing this process by the term paraphyly [20], decided to remove upper-level taxa intending not to represent clustering only exhibited by a few genes as the taxonomic identity of a whole phage genome. To anticipate how ICTV may eventually classify the sequences surrounding LSH1, we examine how they have drawn thresholds in the nearby family *Sarkviridae* related to what is a genus, what is a family, and what is considered no relationship at all. The recently classified family named contains the above-mentioned *Salmonella* phage Jersey, and was first described by the name *Jerseyvirinae* [21], with 3 genera subsequently renamed to *Kaguniavirus*, *Jerseyvirus*, and *Cornellvirus.* Recently, *Guernseyvirinae* was linked to 2 *Serratia* phage genera to form *Sarkvirida*e [27].

Using the ViPTree similarity score axis as an indicator, members of the *Sarkviridae* family have a similarity score over 0.1, members of subfamily *Guernseyvirinae* are over 0.2; and genus members scored greater than 0.3. These thresholds are protein similarity versions of the ANI thresholds recommended by ICTV, but since nucleotide similarity is approaching saturation at the subfamily threshold, we prefer to depend on protein similarity measures. Hence, we expect that, in the official taxonomy, LSH1 will not be joined to the *Sarkviridae* family, and will probably not be linked to the collection of unclassified *Pseudoalteromonas* phages that ViPTree has placed close to LSH1. Furthermore, if the metagenomic sequences used to fill in around LSH1 were subject to ICTV taxonomic assignment consistently with the way *Sarkviridae* was, that collection would produce multiple families. These findings give a quantitative indication of how novel LSH1 is, and of how unevenly sampled the phage world still is.

### AlphaFold studies on the tail end

#### The tail tube

The LSH1 tail tube is encoded upstream of the tape measure as LSH1 gp18 (Fig. 2). Tail tubes are found in 2 varieties, one containing a single tail tube fold with 6 copies forming a ring such as in lambda [28], and one containing 2 tail tube folds with 3 copies forming a similarly shaped ring as in T5 [29]. Siphoviral tail tubes also quite often contain a decoration domain that is positioned on the outside of the ring. In many cases, the decoration has an Ig fold, as it does in lambda and T5. HHpred aligns LSH1 gp18 with the highest scores to dimeric tail tubes, scoring *E* = 5 × 10^−17^ against Protein Data Bank (PDB) ID: 5NGJ_B from T5 [29], but in a pattern indicating that the T5 modification is absent and gp18 has a modification at a different place in the structure. The objective is to clarify what this different decoration is, and whether it is found in other phage tail tubes. The AlphaFold analysis is given in Fig. S3, including matches by the Distance mAtrix aLIgnment (DALI) system, indicating its distribution in phage proteins and other places.

#### The distal tail protein

Downstream of the tape measure gene, LSH1 gp24 is identified as a homolog of lambda gpM distal tail protein by an HHpred alignment *E* = 0.021 with little gapping over the entire tail tube fold, but with a distinct N-terminal domain. The AlphaFold model has the recognizable features of the tail tube fold as described in Fig. S4 with a predicted template modeling (pTM) score of 0.82 and forming the expected 6-fold ring with an interface predicted template modeling (ipTM) score of 0.8. DALI finds the top score with *Z* = 8.1 to lambda gpM, followed by the T5 distal tail protein at *Z* = 7.5. The main point of interest is what the decoration is for which HHpred had nominated a couple of families. TM-align scores on those were obtained as listed in the legend to Fig. S4, finding the best TM-align score of 0.65 to a SCOPE superfamily of galactosidases. A galactose-binding fold has been noted to be found in this position in other siphoviruses [9,15]. The superimposition and TM-align score for the SPP1 member of the family was 0.38 (Fig. S4B). That is on the low end of fold recognition by TM-align, but possibly implicates the LSH1 domain as a divergent member of this family.

#### The tail hub

Downstream of the distal tail protein, there is encoded gp25, the LSH1 hub candidate. The protein encoded after that has credentials to be a side fiber, so apparently gp25 is the entire tail hub assembly. Gp25 has 3 domains based on HHpred matching. The C-terminal domain matches a family of myoviral proteins, most strongly PDB ID: 6F45_D (*E* = 2.9 × 10^−23^), which is the antireceptor on the end of the long fibers of *Salmonella* myovirus S16 [30]. In the myoviral fiber structure, this domain functions as a monomeric polypeptide attached to the end of the otherwise tripartite fiber. The myoviral version is specific for a *Salmonella* outer membrane protein. The mid-section of LSH1 gp25 has HHpred matching to a middle section of lambda gpJ (*E* = 10^−10^) and the N-terminal domain exhibits no HHpred matches. Most puzzling was the absence of HHpred matching to gpL. Also, how the antireceptor domains are configured is of interest.

**Fig. 4.**
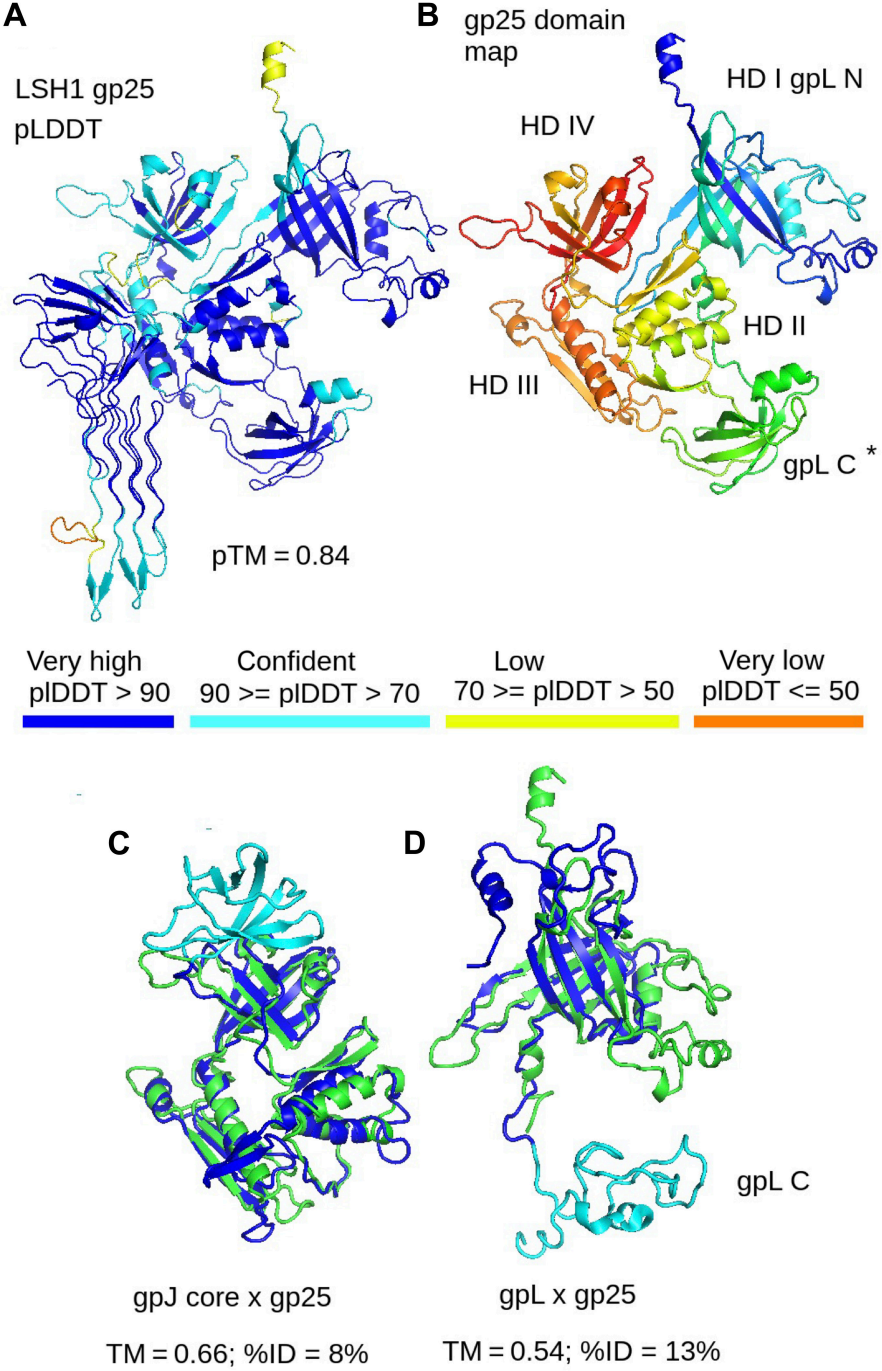
AlphaFold 3 model of the LSH1 hub monomer. (A) Gp25 prediction colored by plDDT. (B) Domain map. The antireceptor domain has been removed. The HD I, II, III, and IV domains are as shown by Wang *et al.* [12]. In lambda, HD II, III, and IV are contained in gpJ. HD I is contained in gpL, which has a C-terminal domain without a domain designation in the HD numbering system. We labeled that gpL C. gpL C* in the LSH1 gp25 structure occupies the same space as lambda gpL, but is a different fold. The actual lambda gpL is seen in the superimposition in panel (D). (C) Superimposition of the homologous portion of gp25 in green on the lambda gpJ core domains in blue, including the Ob decoration in cyan. (D) Superimposition of the homologous portion of gp25 in green on lambda gpL in blue, including its C-terminal domain in cyan.

An AlphaFold 3 model of an LSH1 gp25 monomer is shown in Fig. 4. Figure 4B shows the global domain map used to describe a global sipho- and myoviral hub fold [13], encompassing both the multipolypeptide system of lambda and the single-chain hub as in T5 and LSH1. The LSH1 gp25 N-terminal domain, which HHpred had failed to align with lambda gpL does structurally superimpose with the N-terminal domain of gpL (Fig. 4D), which makes it correspond to the HD I domain in the fused arrangement. LSH1 gp25 then contains a domain that occupies the same space as the C-terminal domain of lambda gpL, which does not have a designation in the HD domain numbering scheme. We designate it as gpL C* in LSH1 because it has a different fold than the actual lambda gpL C domain. After that, the LSH1 chain superimposes with the HD II, HD III, and HD IV domains also found in lambda gpJ (Fig. 4C), missing only the Ob domain found inserted in HD IV in lambda. After HD IV, the chain plunges downwards into the C-terminal antireceptor domain in the same way that lambda gpJ fuses into its large central fiber extension. However, the start of the antireceptor domain sits higher up at what would be the base of the conical tail extension in lambda. In the region occupied by gpL C in lambda and the gpL C* variety in LSH1, there is another small structural protein present in lambda (gpI, not shown), and a much more extensive structure in T5, which is called the “plug” [13]. Lambda has an iron–sulfur cluster in its gpL C domain, although the existence of lambda homologs without the iron–sulfur cluster has been previously reported [31]. The gpL C* region in LSH1 gp25 has no cys residues that could engage in making an iron–sulfur cluster, and its beta sheet structure is unlike anything seen in lambda gpL in this region. In lambda, instead of the N-terminus of the gpJ chain fusing with the C-terminus of gpL as it does in LSH1, it is extended downwards, creating 2 more domains named HDII-ins1 and HDII-ins2, which contribute to the conical appearance of the lambda tail. LSH1 does not have these structures and neither does T5 [13]. *Salmonella* phage Jersey does, so they will appear in the Jersey hub figures below.

**Fig. 5.**
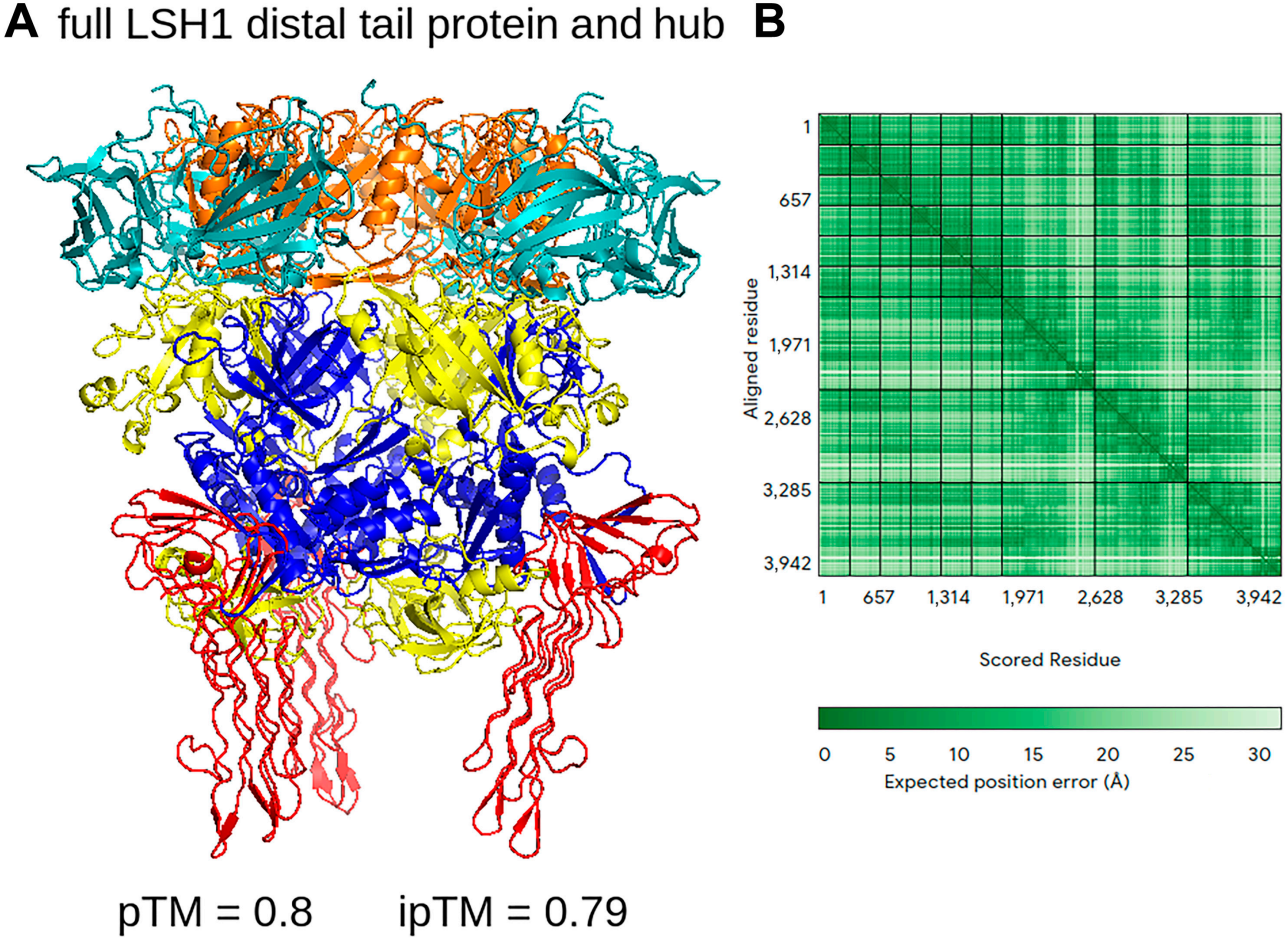
AlphaFold model of the full LSH1 hub. (A) AlphaFold model of 6 distal tail protein (gp24) polypeptides (on top), complexed to 3 hub (gp25) polypeptides. The tail tube domains of gp24 are in orange, with the decorations in teal. In gp25, the domains similar to lambda gpL are in yellow, the domains similar to portions of gpJ are in blue, and the putative antireceptor domains are in red. (B) Position alignment error (PAE) plot.

AlphaFold in multimode was used to assemble 3 copies of LSH1 gp25 together with 6 copies of the distal tail protein, gp24 (Fig. 5). It can be seen that if the antireceptor domains either splay out or rotate to bring the bottom of the tail shaft to the cell wall, contact would be made with the gpL C* region. The packing of the hub against the distal tail protein appears to be similar at least superficially between LSH1 and lambda, although we have not looked at the level of residue contacts.

#### *Salmonella* phage Jersey tail end

**Fig. 6.**
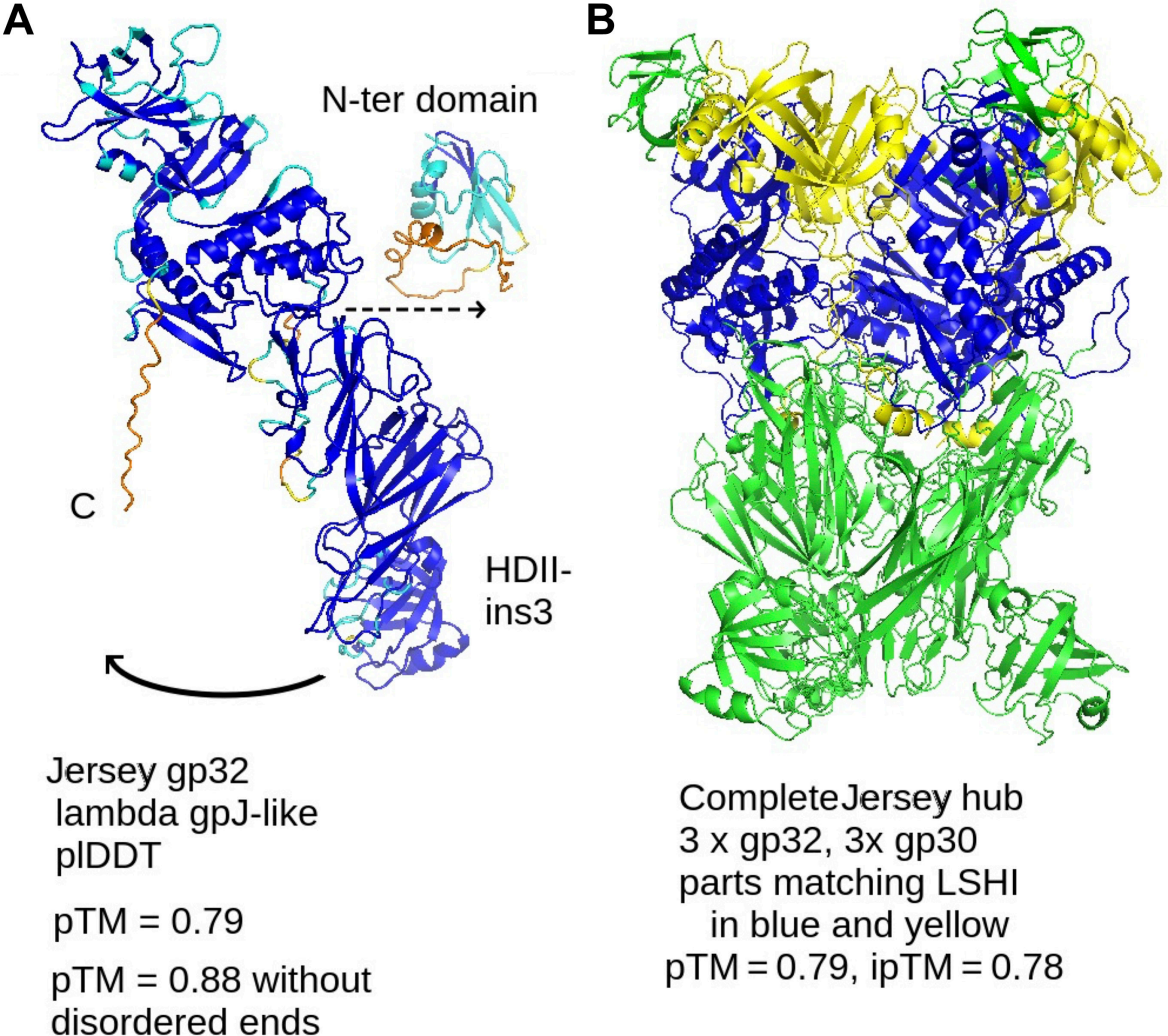
The AlphaFold structure of the Jersey hub. (A) Jersey gp-J like gp32 in plDDT coloration. The details of the N-terminal finger extending down and to the right ending in HDII-ins3 are given in Fig. S5. Panel (A) is oriented according to the domain map in Fig. 4. The novel N-terminal domain has been displaced to the right so as not to hide behind the molecule in this view. (B) Alphafold assembly of the hub with 3 gp32 and 3 gp30. The finger in panel (A) would have to be rotated as indicated to become the front finger in panel (B). Gp30 is a lambda gpL homolog and colored yellow. The part of gpJ-like Jersey gp32 homologous to LSH1 gp25 is colored blue, the remainder is colored green, except that the N-terminal domain has been removed from the figure. For an accounting of that domain and a PAE graph, see Fig. S7.

HHpred predicts that the *Salmonella* phage Jersey hub (Fig. 6) is composed of 2 polypeptides, homologs of lambda gpL (*E* = 1.8 × 10^−14^) and gpJ (*E* = 5.7 × 10^−49^) (Jersey gp30 and gp32, respectively). AlphaFold models are shown in Fig. 6. The Jersey gpJ-like gp32 includes domains like the N-terminal additions to lambda that extend downwards from the hub, and 2 novel additions to that structure as detailed in Fig. S5. One of those, which we will designate as HDII-ins3, is a DALI match at *Z* = 7.1, among many other high-scoring matches, to PDB ID: 9l0f-H in a domain decorating the T1 tail tube and said to have an Ig-like fold [32]. The other is an N-terminal addition that seems to be a small globular domain on a tether for which the position is unclear at this time. Figure 6B shows the multiassembly with the problematic gp32 N-terminal domain omitted. The Jersey gpL-like protein, gp30, is severely truncated on the C-terminal end. The comparison of this region across Jersey, LSH1, and lambda is detailed in Fig. S6. It basically has the tether extending down to the position of the gpL C domain, but then is missing all but one alpha helix. BlastP searching indicates that this arrangement is conserved within *Guernseyvirinae* (not shown). However, in spite of having little to connect to, the tether appears to retain its position in the full hub structure (Fig. 6B and Fig. S6).

The multiconstruction of the Jersey hub, including the problematic gp32 N-terminal region, is shown in Fig. S7. AlphaFold placed the N-terminal region as forming a coiled-coil tether winding up through the lumen with a trimeric domain sitting on top of the hub structure (Fig. S7A, in orange). This is not a plausible position for it, since the lumen of the hub would contain tape measure protein. If given to AlphaFold in isolation, this N-terminal domain does not form a trimer (not shown). If the 3 strands do interact, they would have to be positioned underneath the hub. If they do not interact, they could be splayed out separately to the side. The N-terminal domain did not raise DALI hits. However, a blastP search against other *Guernseyvirinae* genomes shows that this N-terminal sequence is conserved throughout that subfamily, so it presumably must have some functional importance.

#### Side fiber protein

The final LSH1 gene in this structural module, encoding gp26, is in the same genomic position where Jersey encodes its P22-like tail spike. HHpred finds weak (*E* = 1 to 0.0004) matches to a list of tail fibers and tail spikes, but only in short parts of the sequence. Our objective is to make a more definitive description of it, ideally identifying the adhesion or depolymerase domain expected on the C-terminus. Also, because a PsiBlast profile made from Jersey gp32 found 24% identity over 100 residues near the LSH1 gp26 N-terminal region where an attachment domain is expected, and the Jersey fiber is reported to be in 6-fold symmetry, we hoped to exploit that and settle down the attachment site and symmetry of the LSH1 fiber. Symmetry can be important in fiber function. For example, in some phages, it is known that multiple fibers out of 6 have to make contact to trigger ejection [33].

**Fig. 7.**
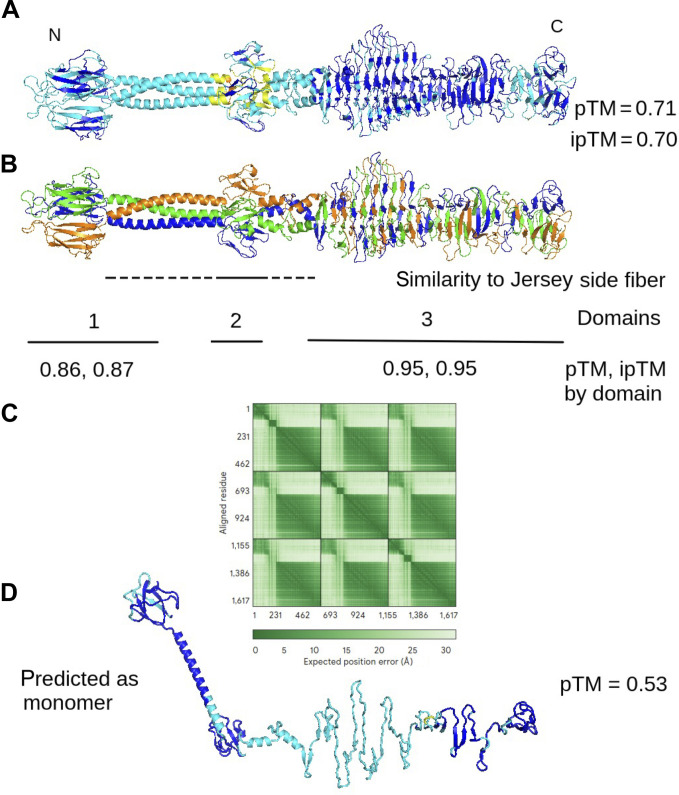
AlphaFold structure of the putative side fiber of LSH1. (A) A triplex of LSH1 gp26 is shown colored by plDDT. (B). The same gp26 triplex structure colored by chain indicating the interdigitated character of the C-terminal domain. Below the structure are indicated 3 domains of interest. Domains 1 and 3, if given to AlphaFold 3 separately in 3 copies, produce the elevated pTM and ipTMs indicated. Domain 2 is the core of a region of similarity with *Salmonella* phage Jersey. When this short sequence is given to AlphaFold 3, it was unable to produce confident structures. However, when this short segment from the triplex models of either the LSH1 protein or the Jersey protein was given to DALI, the same selection of high *Z*-scoring matches was produced (not shown). The dashed line extending the region of similarity with Jersey indicates regions of coiled coil in both phages, although not in a precise structural alignment. (C) PAE plot for the gp26 trimer, (D) The structure given by AlphaFold 3 if instructed to model 1 chain of LSH1 gp26, colored by plDDT score.

The AlphaFold 3 prediction of the side fiber monomer (Fig. 7D) initially appeared difficult to interpret, but when the most common tail fiber stoichiometry of 3 is applied, it becomes clear that AlphaFold was able to anticipate even when only given one copy that this polypeptide was going to be configured as an interdigitated trimer of itself (Fig. 7A and B). DALI searches for the presumptive C-terminal adhesin/depolymerase were at first obscured by matches emanating from the N-terminal region designated domain 2 (Fig. 7B). The domain 2 region is expected to be flexible and is dragging down the pTM for the complex as evidenced by a concentration of low plDDT scores. Making separate predictions for the regions marked domain 1 and 3 in Fig. 7 produced good pTM values, as indicated on the figure, and limiting the DALI search query to just those coordinates allowed good DALI matches for them. Two good matches were found for domain 3. The top DALI hit was a match of the distal portion of LSH1 gp26 to a head fiber of *Pseudomonas* podovirus Pa223 (PDB ID: 9NY6-C; *Z* = 4.5) [34], although it matched only approximately half of the structure. The second hit was a larger match to a protein annotated as a phage-borne hyaluronidase-like protein (PDB ID: 6X3M-F; *Z* = 3.5). Both of these suggest that the LSH1 fiber will be dealing with polysaccharides, not a protein receptor. Although this interdigitated beta helical domain only raised a couple of DALI matches, a survey of phage fibers in PDB found other interdigitated beta helical structure (e.g., PDB ID: 1V0F; PDB ID: 4UXE; PDB ID: 6F45), although less commonly than a trimer of beta barrels, but interdigitated beta helix is common in membrane penetrating “tail spikes” (albeit a different meaning of “tail spike”; e.g., PDB ID: 3VTO, PDB ID: 3QR7, and PDB ID: 1PDL). The DALI matches stemming from domain 2 were to a similar knot-like structure, mostly originating from the N-terminal region of a tail spike from *Escherichia* podovirus CBA120, top match *Z* = 9.1. This is in the region implicated to be similar to the Jersey fiber and expected to be flexible. The PAE plot (Fig. 7C) suggests that it may even be a hinge or a swivel. Domain 2 given to AlphaFold in isolation did not form a valid prediction, but using that region isolated from the full-length coordinate file as a DALI query for both LSH1 and Jersey produced the same spectrum of hits. The LSH1 domain 1 made DALI matches topped by PDB ID: 7eeq (*Z* = 8.5), a tail spike from podovirus Pam1 [35]. The match is to the N-terminal domain of that tail spike, which attaches it to the podoviral tail nozzle. We have thus far had no success in multiconstructs detecting how this fiber joins the LSH1 virion. The Jersey side fiber, although globally like the podoviral P22 tail spike, ends at the beginning of the extended LSH1 similarity indicated in Fig. 7. It has apparently deleted the podoviral attachment domain.

#### Extra structural module

Another structural module encoding LSH1 gp34 to 36 is downstream of the lysis module. The most definitive thing we can say about these genes by preliminary BlastP-based analysis is that among LSH1-related IMGVR genomes, these 3 genes always appear together. However, of all IMGVR sequences that have an LSH1-like hub and side fiber, only half of these have this cluster. That indicates that these 3 proteins work together to make some subassembly, and that they are not essential for hub or side fiber function. The largest of these is gp35, which is examined in Fig. S8. Among named viruses, it exhibits BlastP matches to 2 myoviruses (as evidenced by the presence of a contractile sheath sequence), *Alishewanella* phages Slicko01 and Slickus01 (GenBank OQ508956 and OQ508957). The blast matches are to an N-terminal domain of LSH1 gp34, and it matches 2 and 3 domains in the larger Slicko01 and Slickus01 proteins, respectively. (Fig. S8A). The AlphaFold prediction for gp34 is shown in Fig. S8B. The overall pTM is poor, but the pTM of the N-terminal domain alone is 0.89.

DALI finds matches searching with the whole structure, or the N-terminal domain alone. The top 2 DALI matches are *Z* = 17.5 to PDB ID: 7dmk-A, an alginate lyase [36], and *Z* = 17.1 to PDB ID: 5olr-A, a rhamnogalacturonan lyase [37]. Figure S8D shows a superimposition of the LSH1 gp35 N-terminal domain with the rhamnogalacturonan lyase. Most of the well-overlaid residues are in the beta strands of the beta helix. The helix is indented on one side, identified as the active site in the rhamnogalacturonan lyase. That much appears conserved; however, the extensive surface loops that encompass the substrate polysaccharide in the lyase are much less robust in LSH1 gp34 with generally low confidence plDDT scores. The TM-align score is only 0.45, just below the threshold preferred for fold assignment; however, we never had any expectation that the phage protein and the DALI matches would be specific for the same polysaccharide. A variety of stoichiometries to visualize any higher-order structure with this protein have been tried with no success.

The C-terminal domain of gp34 is modeled as a beta helix, but with statistical indicators in the range where one would have to allow the possibility that it is not a beta helix at all. It straggles off into a disordered chain toward the C-terminus. Poor-quality beta helical predictions also occur for the spacers in and around the conserved gp34-like domains in the myoviral proteins (not shown). AlphaFold is known to produce “hallucinations”, particularly with disordered sequence [8]. Why the predictable domain would be consistently adjacent to long stretches of hallucinatory sequence is not obvious to us. Maybe this sequence has some repetitive motif that would give any multiple alignment algorithm trouble, or maybe it is a linker with so little selection on it that AlphaFold’s usage of covariance fails [38].

Of the 3 proteins, only gp35 raises an HHpred match. It is *E* = 0.0075 against PDB ID: 8JOU_g, which forms a fibril connecting an adhesin to a tail nozzle collar in the podovirus *Ralstonia* phage GP4 [39]. The AlphaFold structure of gp35 is shown in Fig. S9, and it forms a dimer as does the GP4 protein. The AlphaFold structure of LSH1 gp36 is shown in Fig. S9. It has many high-scoring DALI matches that do not particularly suggest any cohesive functional assignment. It appears to be a variation of Ig fold, with 2 antiparallel beta sheets sandwiched at about 20° with the polypeptide chain crossing several times between the 2 sheets. Ig-like folds perform many functions in phages including the collar in GP4; however, we cannot establish a close fold match that would nominate some specific function for gp36, and multiconstructs failed to suggest a partner to which it might bind. Various attempts at multiconstruction failed to build any higher-order structure with gp34, gp35, and gp36.

## Discussion

The isolation of LSH1 from an unusual microenvironment and an unusual host has provided insight into a previously uncharacterized sector of the phage tree. There are metagenomic relatives more closely linked than any currently named phages (Fig. 3). However, the metagenomic relatives, when geolocated, appear to share eutrophic environments as a common feature (Table 1), suggesting that they have maintained a long-term affinity for that environment.

**Table 1.** Isolation sources of the IMGVR sequences in the LSH1-like clade

Genome ID	IMGVR_num	Geographic location	Habitat	Isolation	Latitude	Longitude
Ga0364570_10238	206667	Gwangju, South Korea	Wastewater	Sewage tank	35.09	126.79
Ga0206640_1000216	145405	USA: Illinois, Lake Michigan	Freshwater	Freshwater	41.93	−87.61
Ga0485181_000927	253642	USA: Lake Mendota, Wisconsin	Freshwater	Lake water from pelagic zone	43.10	−89.41
Ga0364484_10620	206260	Seoul, South Korea	Wastewater	Sewage tank	37.58	126.83
Ga0364484_10008	206260	Seoul, South Korea	Wastewater	Sewage tank	37.58	126.83
Ga0207920_1000013	–	–	–	–	–	–
Ga0183686_1000341	183686	USA: Louisiana	Freshwater	River water	29.86	−89.98
Ga0364570_10219	206667	Gwangju, South Korea	Wastewater	Sewage tank	35.09	126.79
Ga0183691_100157	183691	USA: Louisiana	Freshwater	River water	29.86	−89.98
Ga0307966_1000797	307966	USA: Marcell Experimental Forest, Minnesota, USA	Peat soil	Peat soil	47.51	−93.45
Ga0114865_1000184	81084	Western Lake Erie	Freshwater lake	Lake Erie	41.70	−83.26
Ga0451496_0000573	243872	Israel: Golan Heights	Freshwater	River surface water	32.90	35.61
Ga0364481_10091	206257	Seoul, South Korea	Wastewater	Sewage tank	37.56	127.07
Ga0099850_1000056	79182	USA: Chesapeake Bay	Aqueous	Bay water	38.98	−76.37
Ga0114877_102229	81089	Western Lake Erie	Freshwater lake	Lake Erie	41.70	−83.26
Ga0208134_1000129	3300025652	USA: Delaware Bay	Aqueous	Bay water	39.12	−75.26

### Minimal hub structure

Although the LSH1 hub started out with some confusion from HHpred, in the end, it is a reasonably clean representation of the conserved core hub structure. Its only variation from that is that it has 3 relatively small C-terminal domains with credentials to be antireceptors. These domains are also called polyglycine-rich domains or brush domains and have been found in mycobacterial siphoviruses where AlphaFold 2 has been exploited to enumerate variations of tail hub structure [40,41]. Those studies showed marked variation in the structure of the brush domains and means of deployment, emphasizing brush fingers that are glycine-rich and have solvent-exposed patches of hydrophobic residues, presumed to interact with lipid-rich outer cell wall structures. The LSH1 domain shares those features (not shown). LSH1 may provide a simplified model for how the ejection trigger works on gram-negative cell walls. Finding out how widely distributed that polyglycine-rich domain among phages is an interesting problem. We did not find the mycobacterial example with DALI. DALI was blinded by making high-scoring hits to the beta helix of the fold, finding proteins without the brush fingers (not shown). Psiblast searches from the LSH1 sequence found it on hub proteins in *Pseudoalteromonas* phages and some further out than that (not shown), but the oligo glycine stretches and variable number of brush fingers make it a hard target for blast-based software. The polyglycine-rich domain HHpred model of the S16 protein found LSH1 at *E* = 6.9 × 10^−24^, so a forward HMM search might be able to assemble a collection of phage-borne brush domains.

The Jersey hub was brought into the project with the thinking that it might form a clade with LSH1, but structurally, it seems to be only superficially like LSH1. The Jersey hub shares some of the complexity of the lambda hub in terms of keeping some lambda-like domains added to the N-terminus of lambda gpJ. However, Jersey has no C-terminal domain of the kind used as an antireceptor in lambda and T5, or of the kind used by LSH1. Instead, it has added an Ig-like domain to the lambda-like N-terminal extensions in a position that would contact the host cell wall and may therefore be the antireceptor for Jersey. Jersey also has short poorly localized fibers that may be either underneath the hub or splayed out to the side. Those seem to be on a flexible tether, so we do not know a strategy to make AlphaFold localize them. A blastP search of *Guernseyvirinae* proteins (not shown) indicates that this appendage is commonly present in that subfamily, increasing the likelihood that it is doing something. Thus, LSH1 adds 1, and Jersey adds 2 more structural variations to the pantheon of structures appended to siphoviral hubs. The place in the single chain hub that is disrupted by the division of lambda into multiple chains appears to have created a focus of hypervariability, including the aforementioned N-terminal extensions on gpJ and hypervariability of the C-terminal region of gpL. This region is presumably closely juxtaposed to the cell wall.

### Tie in to tree building

AlphaFold 3 multiconstructions were explored to try to firm up certain issues concerning the structural proteins of this new phage LSH1. However, this was done in the service of a long-term objective. We have been working to reintroduce evolutionary trees into the analysis of phage sequences. Using a combination of HMM-based alignments, Bayesian trees, and molecular clock calculation, we have been able to extend lineages of selected phage proteins back into deep time, to explore how they interchange among lineage or descend together, to coordinate the time scale with outside events, such as the mitochondrial endosymbiosis, or the development of host orders and families, and to uncover deep-seated paraphyly in existing phage taxa [17,42,43]. We are also interested in integrating this work with structure-based trees (reviewed in Ref. [44]), which seem likely to extend the analysis to even higher divergence. Both methods require identifying and removing recombination junctions and setting aside domains undergoing extensive modification from the analysis, which can be difficult to do without a structure to see.

The reason to use AlphaFold 3 instead of AlphaFold 2 is that AlphaFold 2 requires a prior alignment, whereas we hope that the structure estimates will guide us through some of the more difficult alignment problems. However, it is also true that we are trying to encourage submitters of new phage sequences to make use of this very available resource and improve the accumulated information about what genes are in these phage families being brought to life by ICTV. The reason to use the multimode is that, while statistical thresholds are important, when trying to recognize a consistent core structure across a set of proteins, there is really no substitute to being able to directly see it and recognize it. If the monomer structure is not enough for that, then seeing it in the context of a subassembly may be. The side fiber (Fig. 7) in this paper is an example of that. In the case of the proteins for which there was ample prior information to know what they should look like, AlphaFold 3 produced recognizable structures with no need for prior alignment, no need to adjust other parameters, and no need to install AlphaFold locally. In the case of the novel 3-protein module, that did not happen. Thus, generally, AlphaFold seems to do well with genes with a lot of preexisting comparative context, but genes without comparative context are still a struggle.

### AlphaFold performance

The AlphaFold performance in this test set could be summarized as follows: Monomer pTMs were generally above the high-quality threshold of 0.8, but chain pTMs in the multiconstructs declined, and declined further with the addition of more polypeptides. We do not know if this is because fitting the interfaces induces more uncertainty, or just some normalization problem based on the number of tokens in the calculation. However, it also happened for the capsomere, portal, and tail tube assemblies of this phage and others, and in working the T5 hub as a control (not shown); thus, we believe it to be systematic. The ipTMs tend to be a little under 0.8, which is considered the high-quality threshold. The training set for these hub complexes has multiple conformations. Some of them undergo a major conformational shift (reviewed in Ref. [10]). All of them have either released the tape measure or still have it, or those forms may even be intermixed in the cryoEM data. Given the conformational variance in the training data, for the ipTMs to be a little light may be inevitable. For the complexes without prior information, trying to find which proteins bind to which by trial and error has thus far been fruitless in our hands.

### Quality thresholds and DALI interpretations

The standard thresholds of quality for AlphaFold are derived by estimating how well the models enable the kinds of analysis structural chemists typically perform using crystal structures. With pTM, it is acknowledged that considering side chain positions is only reasonable with pTM above 0.8, whereas considering backbone configuration is reasonable down to pTM of 0.5. In using TM-align to determine if divergent proteins are “of the same fold”, the gray zone is said to be 0.5 to 0.3, not 0.8 to 0.5 [45]. Thus, different thresholds apply to different applications. For example, noting phage sequences that are not just undergoing simple divergence has become important due to the realization phages often modify proteins with recombination [26], so any sequence that is consistently misbehaving in any alignment program should get attention, not get ignored. A main challenge we see when hunting for homologs with an AlphaFold model and a DALI search is that a boundary is quickly crossed where it is hard to say if the match is to a protein of common ancestry and function, or just to the same fold. A particularly harsh example of this is the aforementioned DALI search with the brush domain producing mostly high scores (*Z* = 7 to 8) to targets that contained beta helices but did not possess the brush fingers (not shown). Similarly, our tail tube decoration and the Jersey HDII-ins 3 both have top scores in the *Z* = 7 to 8 range with plausible phage targets but mixed with yeast enzymes. Thus, it is hard to say that we think the phage proteins have common ancestry and common function when we do not think the yeast enzymes do. Both of those have Ig-like folds. Ig-like folds are so common that chance matching based on general fold only is probably elevated. This is a problem where placing the sequences into a tree may provide some insight by placing the proteins into different clades.

The ambiguity regarding whether a DALI match represents a common fold or common function also affects the decoration on the distal tail protein. It has a fold similar to galectin and even more so to a specific galactoside-binding family, but we cannot know if that is enough to assume it binds galactose. The presence of this domain caused us to wonder why the phage would have any carbohydrate-binding moiety at that site. The distance in the distal tail protein plus hub model (Fig. 4 and Fig. S10) from the distal tail protein decoration to the plane where the lumen of the tail end would meet the bacterial surface is 73 Å. Gram-negative bacteria are covered with lipopolysaccharides, which are very long and highly variable. However, they have specific saccharides in the outer core of the membrane attachment apparatus, which would be 5 to 7 carbohydrate residues up from the bacterial membrane [46], and often there is a galactose there. Carbohydrate residues in a chain are typically about 8.5 Å per residue. Thus, the distal tail protein decorations could be there to bind specific outer core residues that would be located 50 to 68 Å up from the cell wall surface. This would be a tight fit, but a tight fit might be the point by pressing the lumen of the tail tube into the membrane.

### Stoichiometry

On the hub structure, we carried out a retrospective control concerning whether we could have found the number known by prior precedent (3) without that prior information (not shown). The hub was assembled with *n* = 1, 2, 3, or 4 in the presence of the 6-fold distal tail protein ring. The *n* = 1 and 2 models placed the hub subunits against the ring in positions consistent with the *n* = 3 model with no major penalties in ipTM. The space for the missing subunit was simply left blank. However, the *n* = 4 model did create a 4-member ring under the distal tail protein such that reference to the statistical parameters would have to be used to decide between *n* = 3 and *n* = 4 had there been no prior information. In the *n* = 3 model, all chain pair ipTMs fall in the range (0.68 to 0.72). In the *n* = 4 model, chain pair ipTMs between hub and distal tail proteins fall to around 0.15, and hub to hub fall to 0.5 for the adjacent hub, and 0.2 for the hub across the 4-member ring. Thus, in the absence of prior information, it is clear that the correct stoichiometry would have been found as *n* = 3 in this case by looking at chain ipTMs. In hub models without the distal tail protein, the *n* = 2 model gave a false 2-fold symmetry with pTM and ipTM reduced to 0.58 and 0.55, respectively. The *n* = 3 global pTM and ipTM were 0.82 and 0.78, respectively. The *n* = 4 model pTM and ipTM were 0.51 and 0.4, respectively. Thus, although the drop-off in quality was less severe than if assembled with the symmetry match to the distal tail protein, these values would have been sufficient to decide on the *n* = 3 model. However, in controls where proteins with no expectation of binding to the distal tail ring were assembled with it, they generally adopted its 6-fold symmetry. That never gave a result with ipTM above the reject level, but it makes us wary of attempting to prime the assembly of one protein by providing a neighboring assemblage of proteins.

The side fiber presented a very different case in that the monomer was predicted as if it was missing 2 other copies of itself to make an interdigitated beta helix. The trimer prediction matched 3 domains in DALI that are found as part of other trimeric proteins. Thus, in that case, multiple lines of evidence converged to support the presence of a trimeric fiber.

### Domain subdivision

We did not find any case in which dividing the sequence into domains prior to the structure prediction improved the structural prediction, but we did use that strategy post hoc to obtain independent pTM scores for different domains in the protein. This was particularly helpful in the side fiber where there is a region that had generally been expected to be flexible dragging down the global pTM score. What we had not anticipated, but probably should have, is that the domains have to be separated for DALI searches. Otherwise, the top-scoring domain can push the other domains off of the resultant hit list, even if they are high-scoring in their own right.

### The size of things

One thing that the AlphaFold multiconstructions do is give an impression of the relative size and complexity of different subassemblies (Fig. S10). It can be seen that LSH1 has much less at the bottom of the hub than even Jersey. Lambda and T5 have massively larger fibers there (not shown). The size of the poorly understood gp34 protein illustrated in Fig. S10 allows some conception of how much space it may take up when it is finally located. By comparison to the Jersey side fiber, the LSH1 side fiber has much more reach. Given the suggestion that it may have a hinge at the position indicated, it has the appropriate length to form a spider leg-shaped fiber. For the jersey side fiber, the plump shape and the fact that it must be kept close to the hub to contact the cell surface explain why it is much easier to observe by EM.

### Things not accomplished

Our goal to find the site of attachment on the tail shank for the LSH1 side fiber was not achieved. The side fiber clearly has an N-terminal domain homologous to the domain used to attach podovirus tail spikes to the podoviral nozzle protein. Siphoviruses do not have an analog to the podoviral nozzle protein (or at least LSH1 most definitely does not) and AlphaFold 3 did not make a well-predicted complex with anything tested. We did exclude that it might bind through attachment to the novel 3-gene module by showing that the side fiber was present in genomes that did not have that module. The hope that the Jersey side fiber would help did not work out. Although both fibers have components of the P22 side fibers [21], the LSH1 fiber retained the P22-like podoviral attachment domain, whereas the Jersey fiber has lost it (Fig. 7 and Fig. S10). Thus, they attach in 2 different ways, and at this point, we have not clarified how either one of them attaches.

The novel structural module gp34, gp35, gp36 is more an exercise in the bioinformatics objective than a structure/function prediction. We want to pass on to others who hit it in a blastP search what is known about it, rather than having everyone start from scratch trying to annotate it. AlphaFold contributed only a little to that. AlphaFold plus DALI identified a plausible depolymerase/adhesin domain at the N-terminus of gp34. It verified that gp35 found by HHpred to match a fibril protein in GP4 does have the same dimeric structure as that protein. AlphaFold plus DALI indicated that the remaining protein (gp36) has an Ig fold. Thus, the working model is that gp36 attaches the virion to a gp35 fibril, which, in turn, attaches to gp34, which is the payload. AlphaFold did not find evidence of any complex formed among these proteins or binding with any other phage protein. There is a C-terminal domain of gp34 that has very low plDDT values, and we do not know why. We do know that wherever the N-terminal domain is found in other phages, it has something like that poorly predicted domain next to it. Thus, that is a character associated with the protein, even though an unhelpful one at the moment. The AlphaFold structure places a tether between those 2 gp34 domains. We are concerned that if that badly predicted domain is more intricately engaged with the N-terminal domain than simply being attached via a tether, then the relatively high pTM of the N-terminal domain in isolation might be an overestimate. However, the N-terminal domain did appear to have terminal cap visors, which are supposed to prevent that sort of interaction from happening.

## Materials and Methods

### Phage taxa

ICTV taxon names for phages mentioned outside of *Sarkviridae* are *Gervaisevirus* for *Ralstonia* phage GP4; *Trautnerviridae, Polsinellivirinae*, and *Rivavirus* for Bacillus phage SPP1; *Demerecviridae, Markadamsvirinae*, and *Tequintavirus* for *Escherichia* phage T5; *Drexlerviridae, Tunaviridae*, and *Tunavirus* for *Escherichia phage* T1; *Bruynoghevirus* for *Pseudomonas* phage Pa2223; and *Junavirus* for *Lactococcus* phage J-1, also known as *Lactobacillus* phage J-1. Cyanophage Pam1, also known as *Pseudanabaena* phage Pam1, and *Alishewanella* phages Slicko01 and Slickus01 are currently unclassified beyond belonging to *Caudoviricetes* (tailed phages).

### Isolation and growth properties

Two hundred milliliters of SML water was collected with a Harvey rolling drum device [47] from brackish water in tidal Lake Shihwa (37°18′3.6″N, 126°43′58.8″E) located on the west coast of South Korea on 2022 May 5, with an ambient water temperature of 18.9 °C and a salinity of 29.3. For viral isolation, the sample was sequentially filtered through polycarbonate filters with 3.0- and 0.2-μm pore sizes and then concentrated to approximately 500 μl using centrifugal filtration devices (30 kDa Millipore Ultra Centrifugal Filter). Host strains were obtained by incubating on marine agar medium aerobically at 30 °C for 4 days. Strains HL-SH05 and HL-SH06 were isolated and purified via subculturing more than 4 times. For phage LSH1, adsorption assays were performed using mid-exponential-phase host cells (OD₆₀₀ ≈ 0.3; ~1.0 × 10^8^ colony forming units [CFU] ml^−1^) at 30 °C. Unadsorbed phages remaining in the supernatant were quantified by plaque assay, and the adsorption constant was calculated according to standard methods [48]. One-step growth experiments were conducted at 30 °C using a multiplicity of infection of 0.001 and host cell densities of ~1.0 × 10^8^ CFU ml^−1^. After adsorption and removal of unadsorbed phages, samples were collected at 20-min intervals for plaque assays to determine the latent period and burst size. Chloroform sensitivity was evaluated by incubating phage suspensions with 10% chloroform at 30 °C for 1 h. pH and thermal stability assays were performed by incubating phage suspensions under different pH and temperature conditions, respectively, followed by plaque assays to determine residual infectivity. All experiments were independently performed in triplicate.

### TEM observation

Bacteriophage morphology was examined by TEM at the National Instrumentation Center for Environmental Management (NICEM), Seoul National University. CsCl-purified phage particles were deposited onto carbon-coated 200-mesh copper grids pretreated with 0.1% poly-L-lysine for 30 s to enhance particle adsorption. After adsorption for 30 min, the grids were negatively stained with 2% (w/v) uranyl acetate for 30 s. Samples were imaged at 125,000× magnification using a JEM-2100PLUS TEM (JEOL Ltd., Japan) operated at 120 kV.

### Genome sequencing and annotation

Both host genomes and CsCl-purified phage DNA were sequenced with a combination of Illumina MiSeq paired-end short reads and Oxford Nanopore long reads, and assembled and polished with a combination of SPAdes v3.15.5 [49], Unicycler v0.5.0 [50], and Pilon v1.20.1 [51]. After preliminary annotation with Phage Commander [52], open reading frames were reevaluated by a variety of methods previously described [42]. Most relevant to this work were the identification of virion structural proteins by mass spectrometry (see below) and the examination of all reading frames by HHpred [53] (https://toolkit.tuebingen.mpg.de/tools/hhpred; last accessed 2024 June 7). HHpred searches included both the PDB library, and the Uni-Prot-SwissProt-viral70_3_Nov21 library, which integrates HMMs formed from representative phages in the UniProt database into the global collection of searchable HMMs at the HHpred website [54,55]. Matches to PDB entries were further explored by modeling with AlphaFold 3 [8] (https://alphafoldserver.com, accessed 2026 April 20), in some cases followed by structure searches with DALI [56] (http://ekhidna2.biocenter.helsinki.fi/dali/, last accessed 2026 April 19) or structure superimposition by TM-align at the PDB website (https://www.rcsb.org/alignment, last accessed 2026 April 25) [45].

### AlphaFold

AlphaFold 3 [8] was used as follows. Monomers were put in the Google Deep Mind server (https://alphafoldserver.com; last accessed 2026 April 25). Default templates were left in place. No specific multiple sequence alignments or templates were added. We did not make multiple predictions with different seeds and pick the best one for presentation. Multiple predictions of subassemblies were made first with polypeptides and stoichiometries drawn from prior expectations. The use of AlphaFold 3 in multimer mode to expose any previously unsuspected interaction has yet to yield a useful result for us. In some cases, predictions were repeated with wrong stoichiometries to see if the statistics reported could have independently led us to a particular stoichiometry. Polypeptides were not sectioned into domains in advance. In some cases, where the pTM was low but the predicted local distance difference test (plDDT) indicated that there were one or more well-predicted domains, a prediction was conducted on that section of sequence in isolation to discover what its pTM would be if not dragged down by neighboring domains. Discussion of statistical quality is based on recommendations from the EMBL-EBI AlphaFold tutorial (https://www.ebi.ac.uk/training/online/courses/alphafold/#vf-tabs_section--contents; accessed 2026 April 16). From high to low quality, 3 categories of pTM have thresholds at 0.8 and 0.5 (out of 1.0), 3 categories of ipTM have thresholds at 0.8 and 0.6, and 4 categories of plDDT have thresholds at 90, 70, and 50 (out of 100). Note that pTM is a prediction of the TM-align score of the model to the crystal structure of the same protein if it were available. TM-align is used to determine if divergent structures are related, with down to 0.5 interpreted as of the same fold, and 0.2 to 0.5 as maybe of the same fold or parts of it have the same fold. Structure figures were rendered with the PyMOL Molecular Graphics System, Schrödinger, LLC.

### Mass spectrometry

Mass spectrometry was conducted at NICEM, Seoul National University. The RP-nano LC-ESI-MS/MS analysis was performed using a Thermo Scientific Q Exactive Hybrid Quadrupole-Orbitrap instrument (Thermo Scientific, San Jose, CA, USA) equipped with Dionex U 3000 RSLCnano HPLC system. Assignment of mass spectra to phage proteins was carried out using Proteome Discovery software (Thermo Scientific). Mass spectrometric results are shown in Table S1, including spectrum counts/MW as a crude indication of abundance. The inferred virion proteins are also indicated in Fig. 2. Sensitivity was sufficient to detect the presumably substoichiometric scaffold protein. No viral proteins of an obvious nonstructural nature were detected.

### ViPTree analysis

The ViPTree server [57] (https://www.genome.jp/viptree/; accessed 2025 May 21) concatenates protein sequences detected by TblastX and constructs a clustergram together with its database of previously analyzed sequences. The NCBI taxonomy browser [58] was used to associate previously established ICTV taxon assignments of the clusters most closely linked to the LSH1 query. In Fig. 3, the very large *Guernseyvirinae* subfamily was manually collapsed to one branch per genus. Metagenomic sequences from IMGVR [25] were included as follows. The IMGVR database was imported and screened with TblastX against the LSH1 sequence. A total of 435 sequences were chosen based on the criterion of being at least 20 kb with at least 2,000 residues in alignment with LSH1, and a TBlastX similarity score comparable to or better than that between LSH1 and the closest *Pseudoalteromonas* phages. Trying to add that many sequences to the ViPTree server overpowered it; thus, the algorithm ViPTreeGen was installed locally (obtained 2025 March 19) and run with the 435 sequences and a selection of phage sequences previously mapped by ViPTree around LSH1. Of these sequences, 75 were clustered with LSH1 inside of the linkage to the *Pseudoalteromonas* phages. A set of 16 of those chosen to represent the greatest diversity of the set, as well as the closest sequences linked to LSH1, were sent together with LSH1 to the ViPTree server to make the tree shown in Fig. 3. Of those 75 IMGVR sequences, 68 had annotated reading frames. A database of those was compiled for BlastP searching to ascertain how consistently features found in LSH1 were conserved in the IMGVR-defined neighborhood.

## Data Availability

The sequence and annotation of *Alishewanella* phage LSH1 were deposited in GenBank as accession PX020959. The sequences of *Alishewanella* sp. strains HL-SH05 and HL-SH06 were deposited as accessions CP199753 and CP199752. The LSH1 hub model is available in ModelArchive at https://www.modelarchive.org/doi/10.5452/ma-ytywj.
