# Supplementary material for: Alishewanella Phage LSH1 from the Sea Surface Microlayer Provides a Novel Minimalistic View of the Siphoviral Hub Structure
Source: Comput Struct Biotechnol J. 2026 Jun 8;35(1):0131. doi: 10.34133/csbj.0131 (PMC13243795; doi:10.34133/csbj.0131)
Supplement: Supplementary 1 — Figs. S1 to S10 Tables S1 and S2 [file csbj.0131.f1.pdf]

TABLE S1. Mass spectrometry data for *Alishewanella* phage LSH1 virions

Name	Product	Coverage	SC	Uniq. pep	MW	SC/MW	pI
LSH1 gp7	minor head pr. SPP1 gp7-like	38	32	11	41	0.78	9.16
LSH1 gp8	portal	38	43	14	54.7	0.79	4.77
LSH1 gp9	scaffold	4	1	1	25.3	0.04	5.66
LSH1 gp10	MCP	54	171	12	34.2	5.00	5.45
LSH1 gp12	gp12	42	31	6	20.9	1.48	5.2
LSH1 gp14	head completion SPP1 gp15-like	59	16	5	15.1	1.06	5.05
LSH1 gp15	head completion lambda FII-like	37	12	3	13.4	0.90	4.63
LSH1 gp16	tail term. plug HK97 gp10-like	10	1	1	13.1	0.08	4.56
LSH1 gp17	tail term. SPP1 gp17-like	51	13	5	15.5	0.84	4.81
LSH1 gp18	tail tube	37	53	10	41.4	1.28	4.74
LSH1 gp23	tape measure	47	109	27	83.3	1.31	5.19
LSH1 gp24	distal tail pr. lambda gpM-like	21	9	4	28.3	0.32	5.21
LSH1 gp25	hub pr. lambda gpJ-like	17	25	9	83.4	0.30	4.93
LSH1 gp26	side fiber	44	51	15	56.6	0.90	5.02
LSH1 gp34	putative adhesin	9	3	2	50.1	0.06	6.04
LSH1 gp35	putative connector	19	16	2	14.3	1.12	4.94
LSH1 gp36	putative collar	54	16	4	11.1	1.44	4.22

TABLE S2. IMGVR sequences in the LSH1-like clade

IMGVR_UViG_3300025687_000156|3300025687|Ga0208019_1000181
IMGVR_UViG_3300007960_000057|3300007960|Ga0099850_1000062
IMGVR_UViG_3300048672_000148|3300048672|Ga0485282_000207
IMGVR_UViG_3300046011_003262|3300046011|Ga0485186_0000211
IMGVR_UViG_3300033120_000132|3300033120|Ga0364481_10004
IMGVR_UViG_3300046005_000806|3300046005|Ga0485180_000227
IMGVR_UViG_3300024979_000010|3300024979|Ga0208115_1000046
IMGVR_UViG_3300024976_000001|3300024976|Ga0208628_1000172
IMGVR_UViG_3300004179_000005|3300004179|Ga0066404_1000072
IMGVR_UViG_3300004173_000004|3300004173|Ga0066412_1000129
IMGVR_UViG_3300033120_000242|3300033120|Ga0364481_10001
IMGVR_UViG_3300033068_000382|3300033068|Ga0364484_10008
IMGVR_UViG_3300031607_000017|3300031607|Ga0307966_1000797
IMGVR_UViG_3300017754_001608|3300017754|Ga0181344_1000079
IMGVR_UViG_3300031965_000090|3300031965|Ga0326597_10001572
IMGVR_UViG_3300033068_000645|3300033068|Ga0364484_10016
IMGVR_UViG_3300033068_000170|3300033068|Ga0364484_10363
IMGVR_UViG_3300019763_000002|3300019763|Ga0193962_1000080
IMGVR_UViG_3300030390_001624|3300030390|Ga0183691_100157
IMGVR_UViG_3300033068_000800|3300033068|Ga0364484_10620
IMGVR_UViG_3300001125_000001|3300001125|JGI11931J13222_1000002
IMGVR_UViG_3300046008_000424|3300046008|Ga0485183_000161
IMGVR_UViG_3300046007_003831|3300046007|Ga0485182_000275
IMGVR_UViG_3300046006_002185|3300046006|Ga0485181_000927
IMGVR_UViG_3300035703_000194|3300035703|Ga0310140_0001836
IMGVR_UViG_3300034110_000260|3300034110|Ga0335055_0000327
IMGVR_UViG_3300020036_001055|3300020036|Ga0206648_1001134
IMGVR_UViG_3300020042_006894|3300020042|Ga0206640_1000216
IMGVR_UViG_3300033072_000183|3300033072|Ga0364570_10238
IMGVR_UViG_3300020044_005149|3300020044|Ga0206656_1000138
IMGVR_UViG_3300030100_001363|3300030100|Ga0183667_1000309|5935-39406
IMGVR_UViG_3300030406_007280|3300030406|Ga0183721_1000007|157692-199942
IMGVR_UViG_3300030392_002194|3300030392|Ga0183717_1000145|14403-56653
IMGVR_UViG_3300037195_002098|3300037195|Ga0183732_000006|227635-269885
IMGVR_UViG_2806310755_000001|2806310755|2806358958|18049-60299
IMGVR_UViG_3300030059_002546|3300030059|Ga0183686_1000341
IMGVR_UViG_2788500080_000001|2788500080|2788533675
IMGVR_UViG_3300033069_000234|3300033069|Ga0364485_10092
IMGVR_UViG_3300048672_000344|3300048672|Ga0485282_000203
IMGVR_UViG_3300033120_000309|3300033120|Ga0364481_10111
IMGVR_UViG_3300038314_000845|3300038314|Ga0183735_000073
IMGVR_UViG_3300037785_000033|3300037785|Ga0394794_000394
IMGVR_UViG_3300020044_005099|3300020044|Ga0206656_1000300
IMGVR_UViG_3300033072_000576|3300033072|Ga0364570_10219
IMGVR_UViG_3300020047_006439|3300020047|Ga0206650_1000282
IMGVR_UViG_3300008453_000582|3300008453|Ga0114877_102229

IMGVR_UViG_3300008448_000223|3300008448|Ga0114876_1000272
IMGVR_UViG_3300033120_000088|3300033120|Ga0364481_10091
IMGVR_UViG_3300042260_000229|3300042260|Ga0451496_0000573
IMGVR_UViG_3300008462_000493|3300008462|Ga0114866_101544
IMGVR_UViG_3300008459_000141|3300008459|Ga0114865_1000184
IMGVR_UViG_3300008267_000135|3300008267|Ga0114364_1000174
IMGVR_UViG_3300025652_000011|3300025652|Ga0208134_1000129
IMGVR_UViG_3300025645_000015|3300025645|Ga0208643_1000153
IMGVR_UViG_3300022178_000006|3300022178|Ga0196887_1000068
IMGVR_UViG_3300007276_000014|3300007276|Ga0070747_1000068
IMGVR_UViG_3300006920_000013|3300006920|Ga0070748_1000090
IMGVR_UViG_3300028423_000317|3300028423|Ga0307028_1000043
IMGVR_UViG_3300035698_008418|3300035698|Ga0374944_609224
IMGVR_UViG_3300047557_002176|3300047557|Ga0485255_000211
IMGVR_UViG_3300044825_001500|3300044825|Ga0451703_0000213
IMGVR_UViG_3300001605_000264|3300001605|Draft_10002092
IMGVR_UViG_3300025655_000011|3300025655|Ga0208795_1000094
IMGVR_UViG_3300025647_000007|3300025647|Ga0208160_1000083
IMGVR_UViG_3300025646_000003|3300025646|Ga0208161_1000115
IMGVR_UViG_3300022200_000010|3300022200|Ga0196901_1000113
IMGVR_UViG_3300022198_002025|3300022198|Ga0196905_1000048
IMGVR_UViG_3300007960_000006|3300007960|Ga0099850_1000056
IMGVR_UViG_3300007542_000009|3300007542|Ga0099846_1000050
IMGVR_UViG_3300007541_000005|3300007541|Ga0099848_1000048
IMGVR_UViG_3300007538_000009|3300007538|Ga0099851_1000054
IMGVR_UViG_3300021197_001246|3300021197|Ga0219031_10332
IMGVR_UViG_3300025218_000002|3300025218|Ga0207882_1000104
IMGVR_UViG_3300001726_000001|3300001726|JGI24653J20064_1000090
IMGVR_UViG_3300025687_000012|3300025687|Ga0208019_1000117

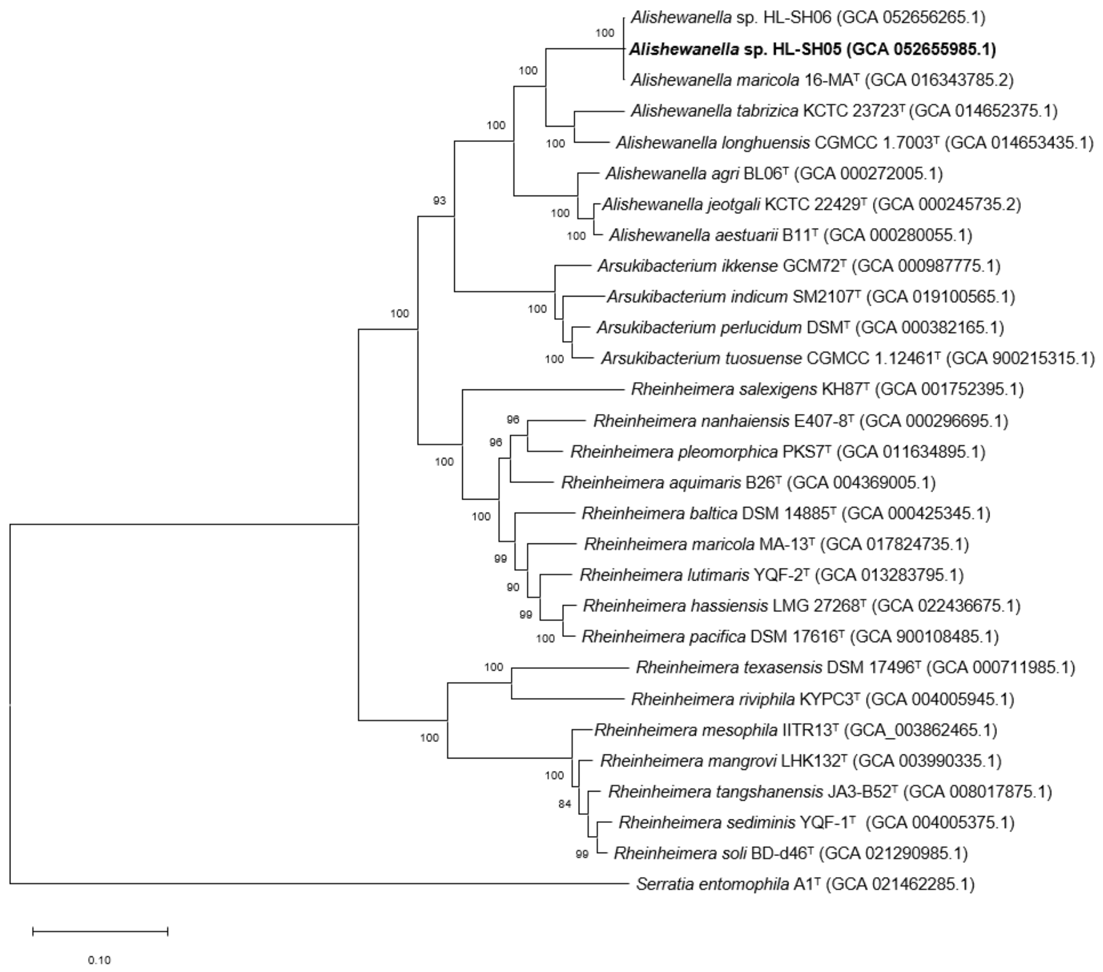

FIG. S1. Maximum-likelihood phylogenomic tree based on amino acid sequences of 120 concatenated marker genes showing the phylogenetic relationship of strain HL-SH05 to closely related members of the genus *Alishewanella*. Percentage bootstrap values above 70 % (1000 replicates) are shown at branch nodes. *Serratia entomophila* A1^{T} was used as an outgroup. Scale bar represents 0.10 amino acid substitutions per site.

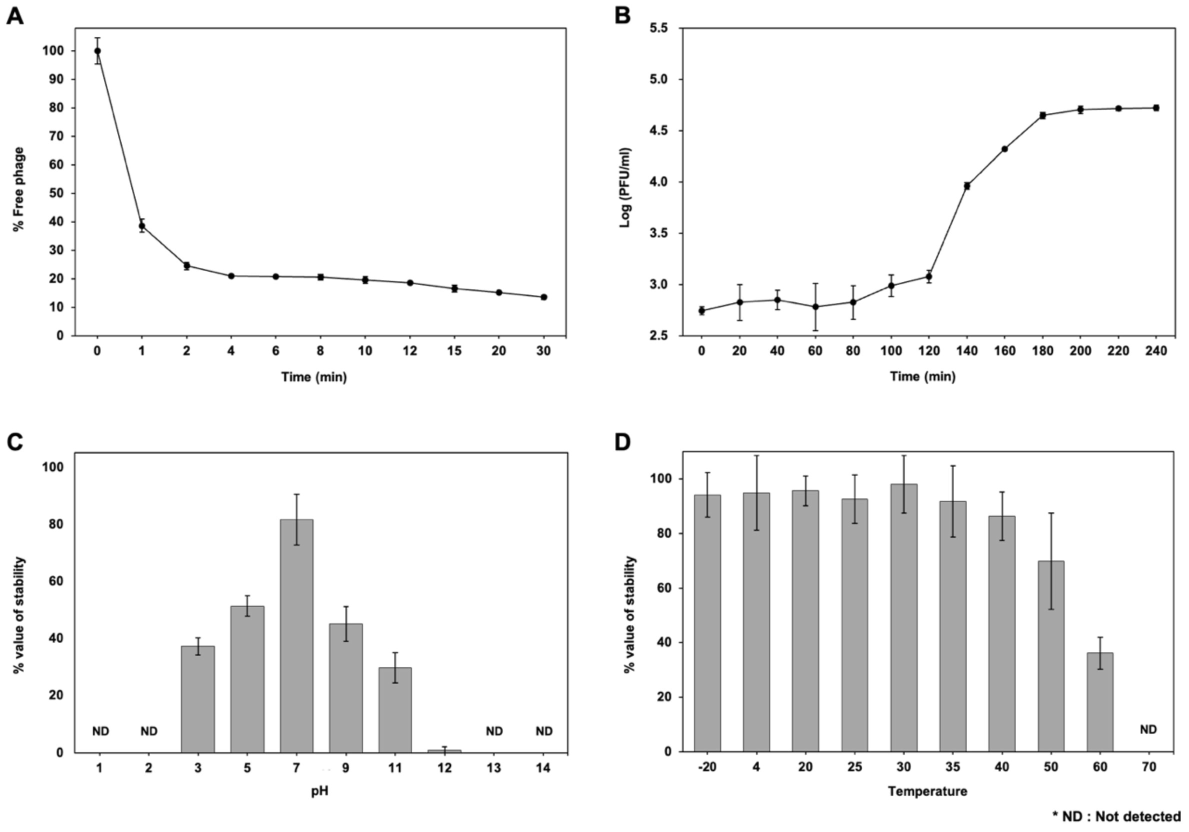

FIG. S2. Growth properties of *Alishewanella* phage LSH1. A) Adsorption curve yielding an adsorption constant of 6.7 x 10^{-9}. B) One-step growth curve yielding a latent period of 120 min and a burst size of 68 ± 7 at 30 ºC. C) pH profile – survival at 30 ºC after 1 hour. D) Thermal stability after 1 hour. Data are presented as mean ± SD from three replicates. ND, not detected.

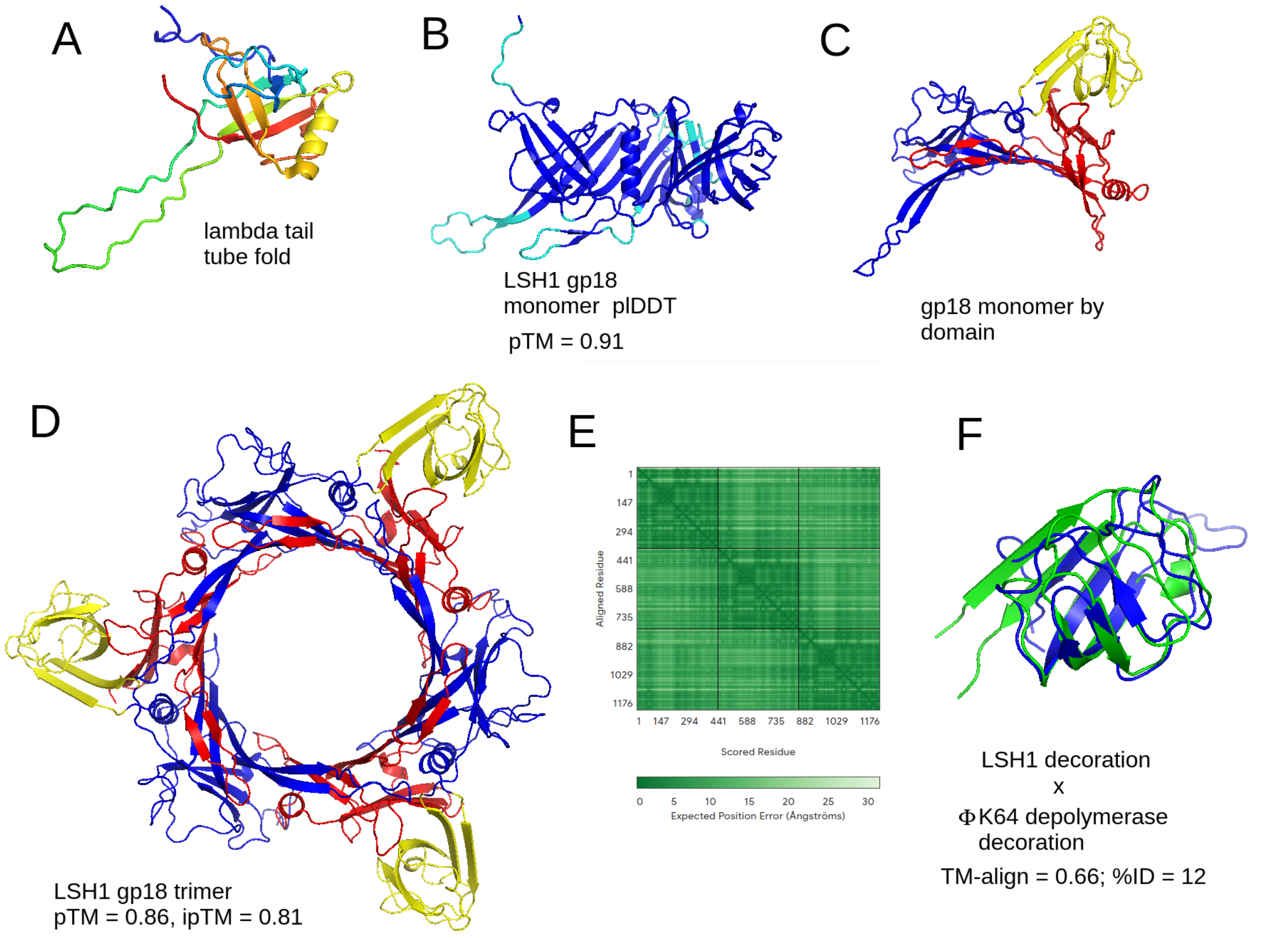

FIG. S3. AlphaFold modeling of LSH1 gp18 tail tube. A) The canonical tail tube fold illustrated by pdb:6p3e [1] with 2 antiparallel sheets, an alpha helix, and a prominent loop extending beneath the adjacent subunit in a six-member ring. Colored blue -> red, N -> C B) LSH1 gp18 monomer with two tail tube domains colored by plDDT (see Fig. S4 for scale). C) LSH1 gp18 monomer viewed from the tail end colored by domain: N-terminal domain - blue, decoration inserted after the helix in domain 1 - yellow, C-terminal domain - red. D) One LSH1 gp18 tail tube ring with the same color code and orientation as C. E) PAE plot for panel C. F) Superimposition of the LSH1 decoration (green) on pdb:9k4a-B *Klebsiella* phage K64-1 depolymerase Z = 8.0. DALI found many high-scoring matches to this domain with the strongest to a domain in Ubiquitin-activating enzyme E1, e.g., pdb:3cmm-A, Z=8.1, and many phage tail spikes and appendages with the strongest being *Klebsiella* phage K64-1 depolymerase, where it decorates a structure composed of a trimer of beta helices, and is thought to stabilize the trimeric structure [2].

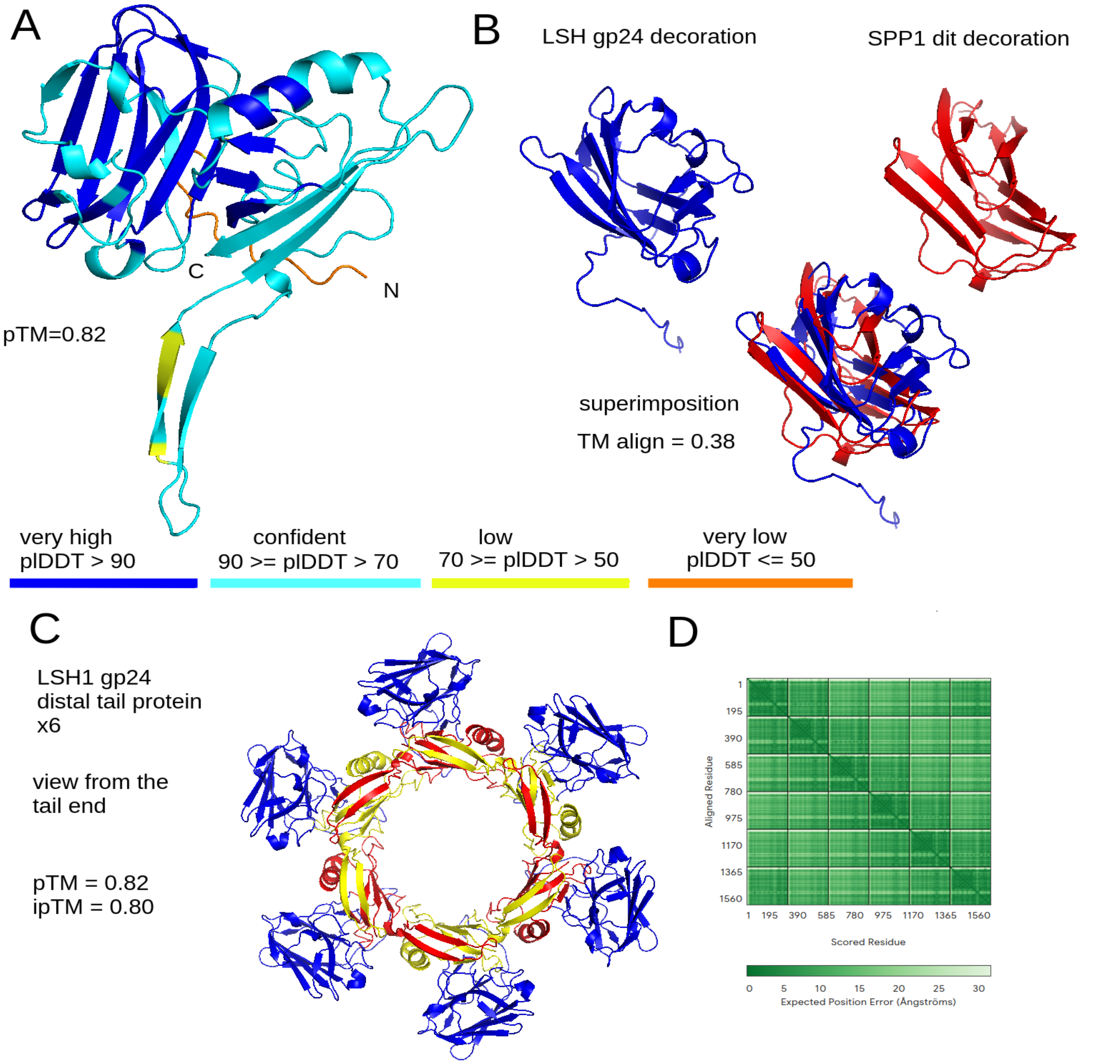

FIG. S4. AlphaFold model of the six-fold ring of LSH1 gp24, distal tail protein. There are six polypeptides, each with a tail tube fold, and each with an N-terminal decoration (residues 1-151). A) Monomer colored by plDDT. B) Superimposition of the LSH1 decoration with the SPP1 galectin decoration. C) Six-fold ring colored by domain, tail tube fold alternating red and yellow, decoration is in blue. HHpred matched the decoration to glycosylase structures, and particularly galactosidases (pdb:1k3i, galactosidase superfamily, TM align =0.65, %ID=11%; pdb:6MDS, TM align = 0.53; %ID 8%). D) PAE

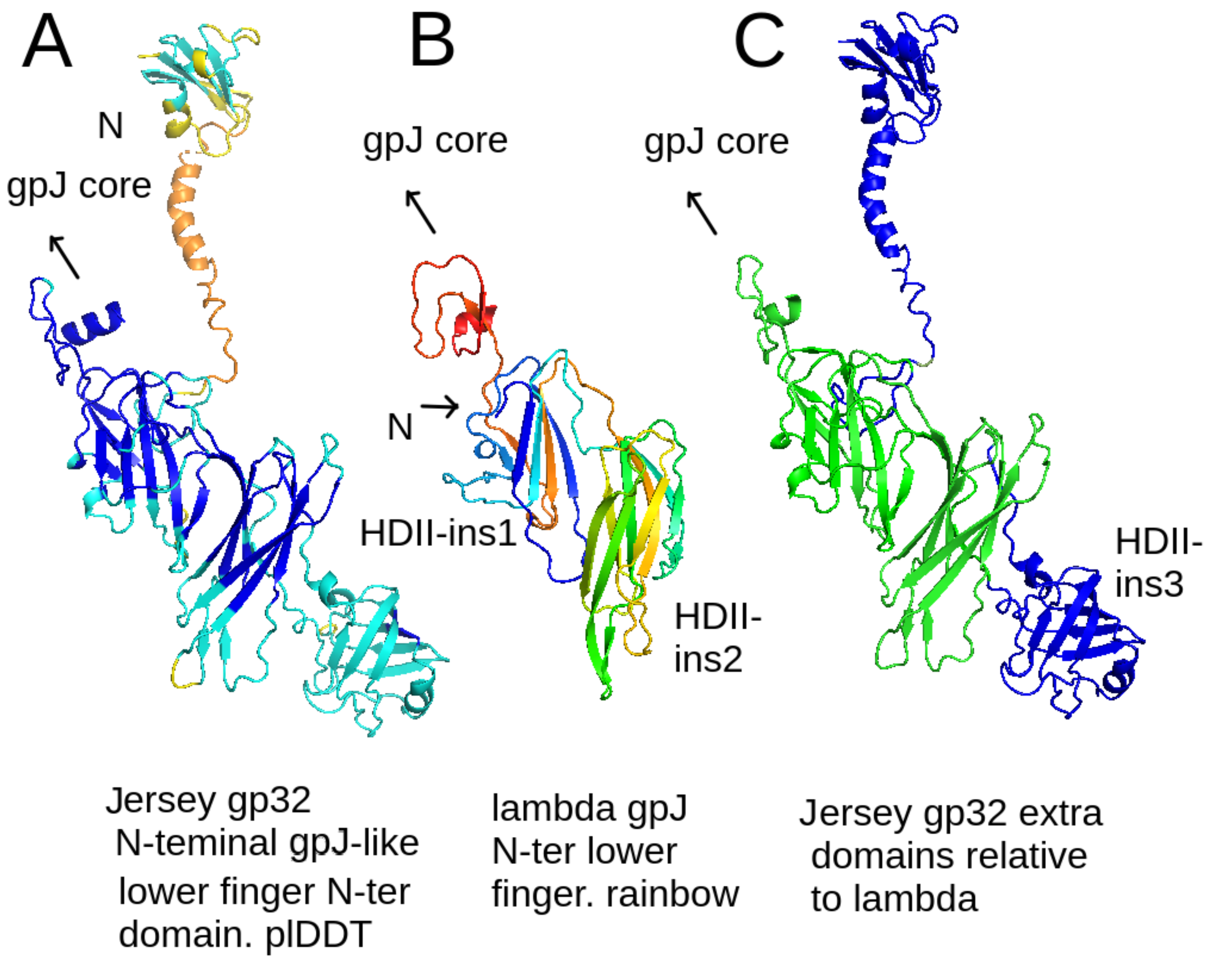

FIG. S5 AlphaFold prediction of the structure of the Jersey N-terminal finger on gpJ-like Jersey gp32. A) predicted structure of the N-terminal extension of Jersey gp32 colored by plDDT. None of this sequence is present in LSH1. It is an elaboration of the N-terminal finger on lambda gpJ. B) Domain map of the N-terminal finger on lambda gpJ in blue -> red - N->C. The two domains named HDII-ins1 and HDII-ins2 are as described [3,4]. C) domain map of Jersey gp32. The parts homologous to lambda are in green. Two new domains have been added. An Ig domain has been added at the end of the finger, designated HDII-ins3. The N-terminus has been extended to a small globular domain. The direction of the extension is unknown, as indicated by the orange color of the plDDT map.

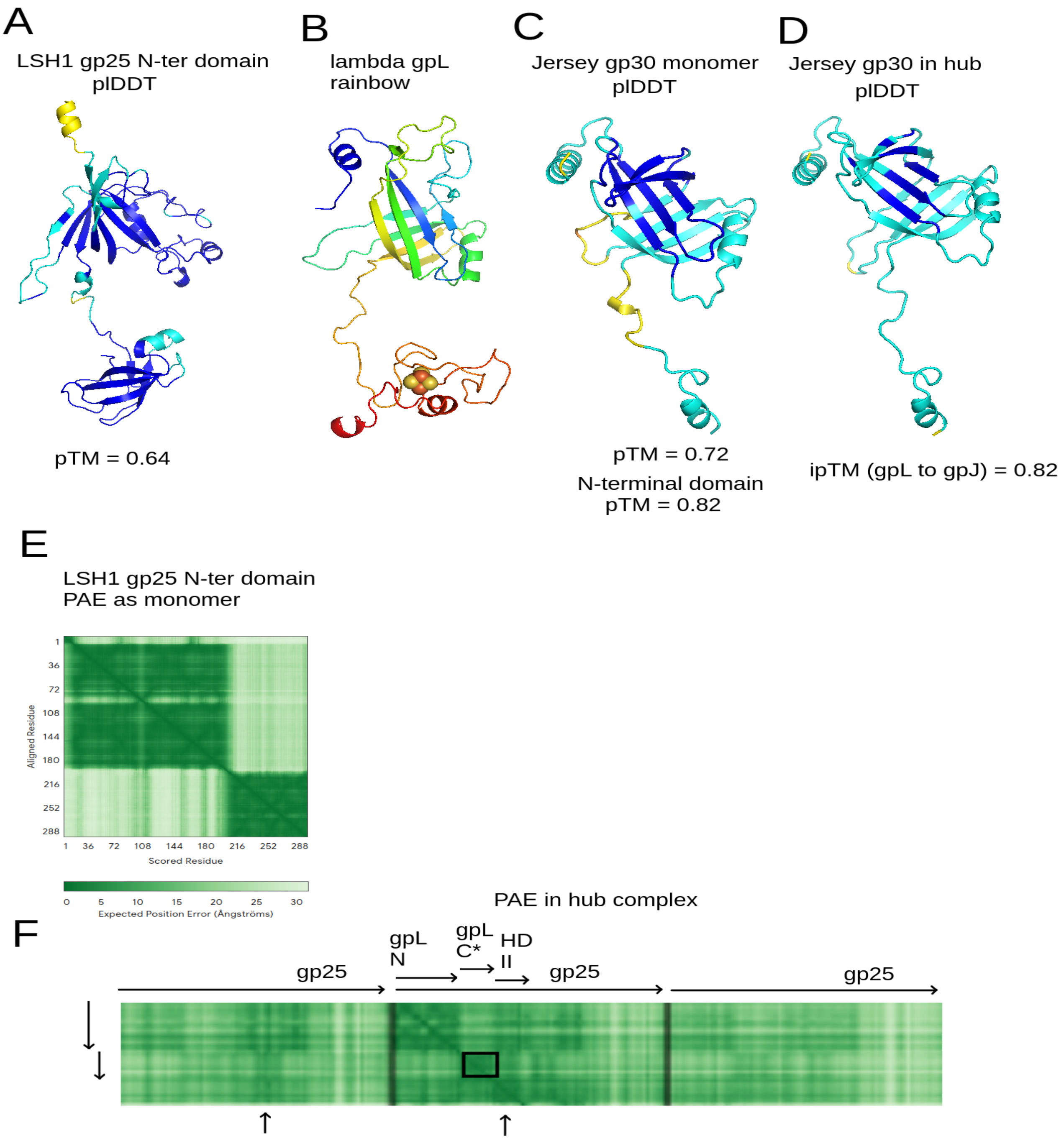

FIG. S6. Comparison of LSH1 gp25 lambda gpL-like domain with lambda gpL and Jersey gp30. A) AlphaFold model of the N-terminal domain of LSH1 gp25 (gpL N and gpL C*) modeled in isolation and colored by plDDT. B) lambda gpL for pdb:8k35 blue->red, N->C. C) Jersey gp30 monomer AlphaFold model colored in plDDT, indicating that the main conformational uncertainty is the placement of the C-terminal domain relative to the N-terminal domain. D) improved confidence in the configuration of the gpL N to C connector when assembled into the hub. E) PAE of the gp25 N-terminal domain in isolation, indicating the main uncertainty is placement of the gpL C* domain. F) Section of PAE from the fully assembled hub showing that the gpL C* domain is more integrated with the HDII domains of its own polypeptide and of a neighboring subunit than it is to its own N-terminal domain or other gpL C* domains in the hub.

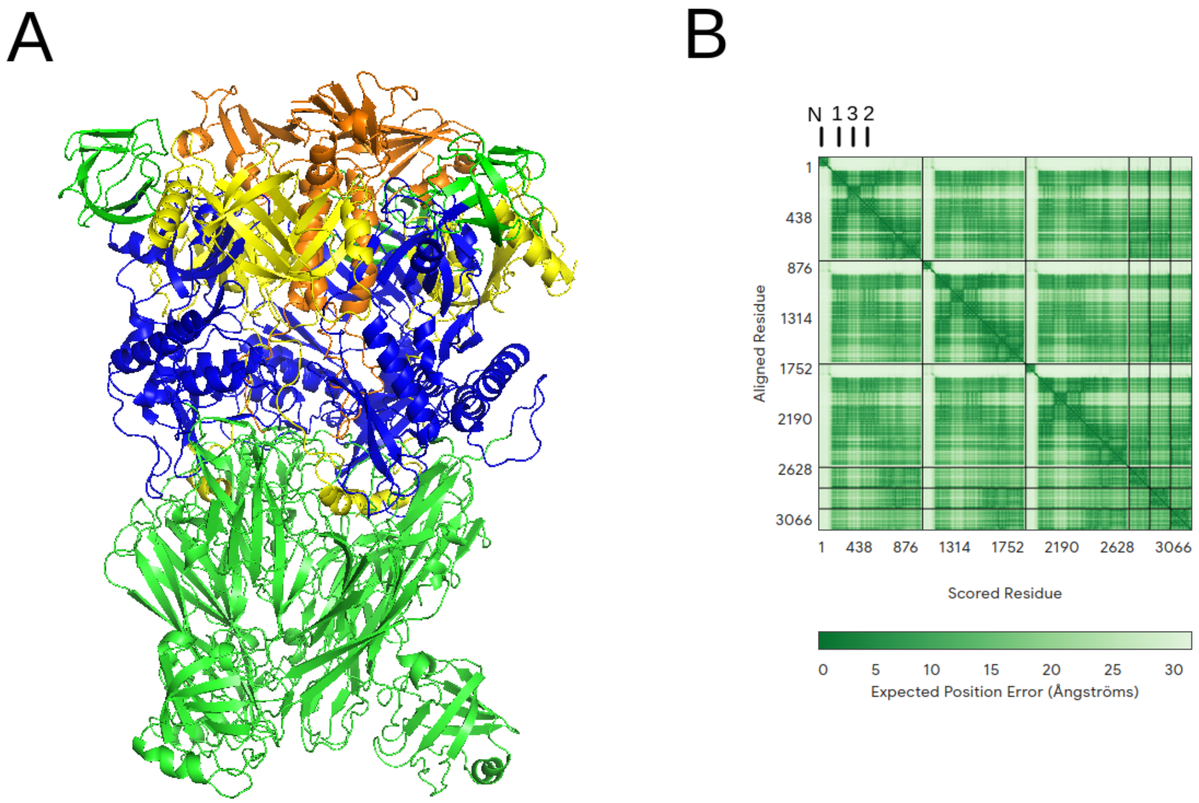

FIG. S7. Full AlphaFold model of the Jersey hub. A) Jersey hub formed by 3 copies of gp32 and 3 copies of gp30 colored as in Fig. 6 except the implausibly located gp30 N-terminal domain is present and indicated in orange. B) PAE, with the location of the domains as described in Fig. 5. N is the (orange) N-terminal domain. 1, and 2 are the lambda-like N-terminal HDII-ins1 and HDII-ins2 domains. 3 is the HDII-ins3 Ig-like domain new in the Jersey fold.

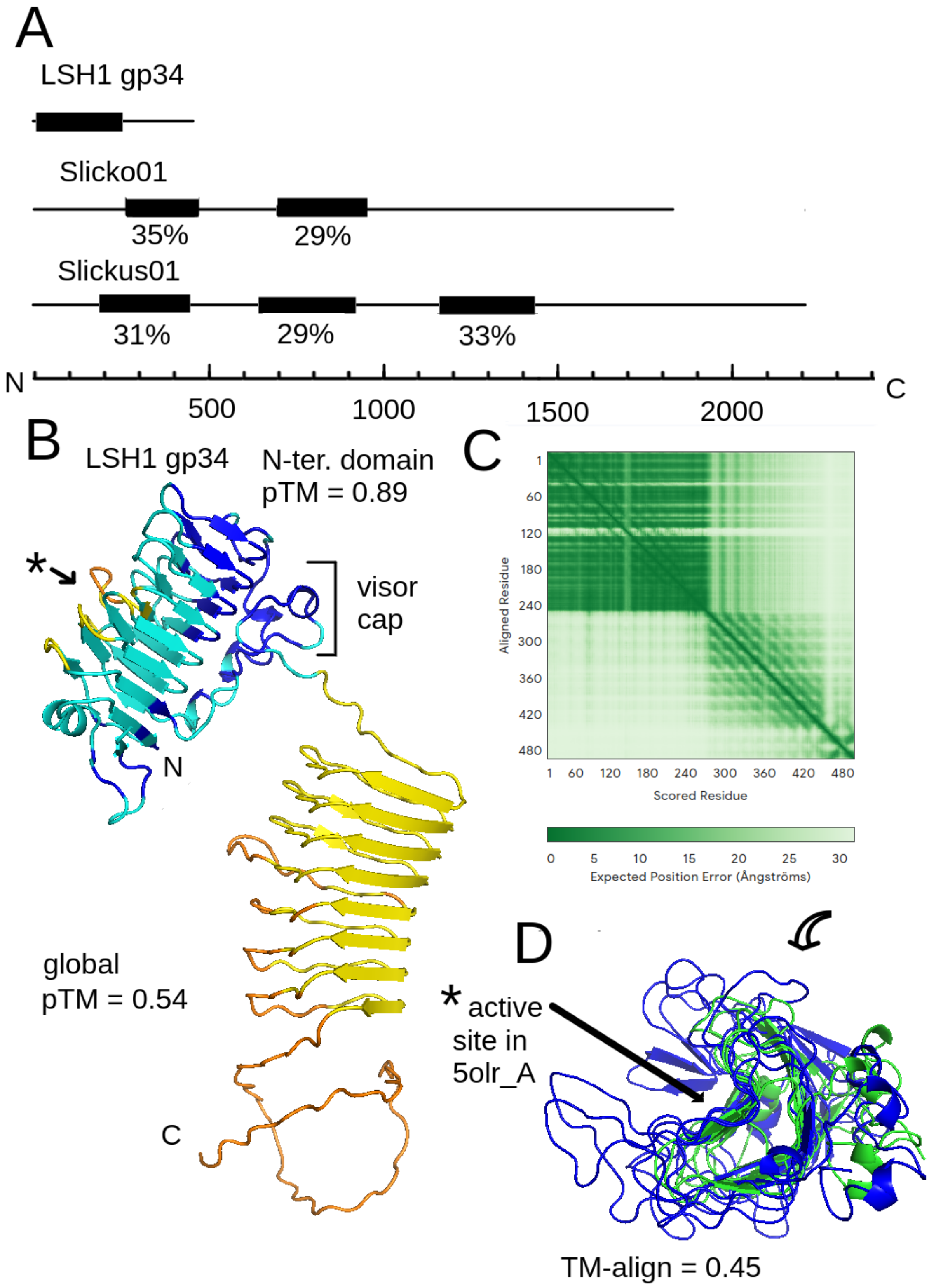

FIG. S8. BlastP and AlphaFold 3 analysis of LSH1 gp34, and related proteins in vB_AspM_Slicko01 and vB_AspM_slickus01. A) BlastP matching of the conserved N-terminal domain of LSH1 gp34 to multiple domains in the two myoviruses. The Slicko01 protein is accession WGH49683 and Slickus01

is WGH49876. Percent identity is given under each matching domain. The scale is in the number of residues. B) AlphaFold 3 prediction of LSH1 gp34 colored to represent plDDT. The visor cap refers to a structure described [4,5] to prevent the concatenation of beta helical domains. C) PAE plot. D) Superimposition using TM align of LSH1 gp34 N-terminal domain versus pdb:5olr-A [6]. The beta helix is tilted down to look through the groove, which contains the active site in 5olr. * indicates the position corresponding to that site in panel D and B. blue - pdb:5olr; green - LSH1 gp34 N-terminal domain.

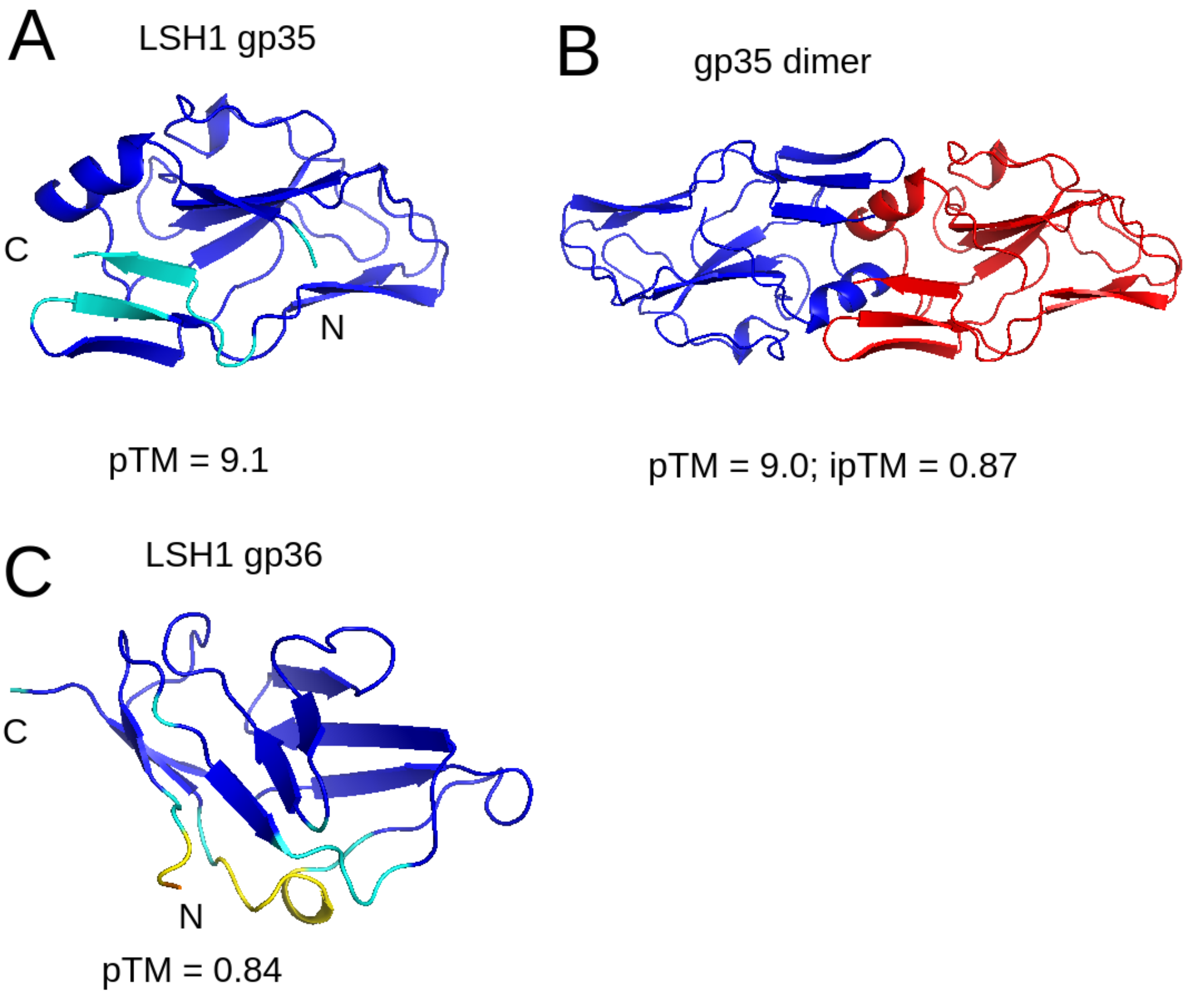

FIG. S9. LSH1 gp35 and gp36 AlphaFold models
A) AlphaFold model of gp35 colored according to plDDT. B) Dimer model of gp35 colored by chain.
C) AlphaFold model of gp36 colored according to plDDT.

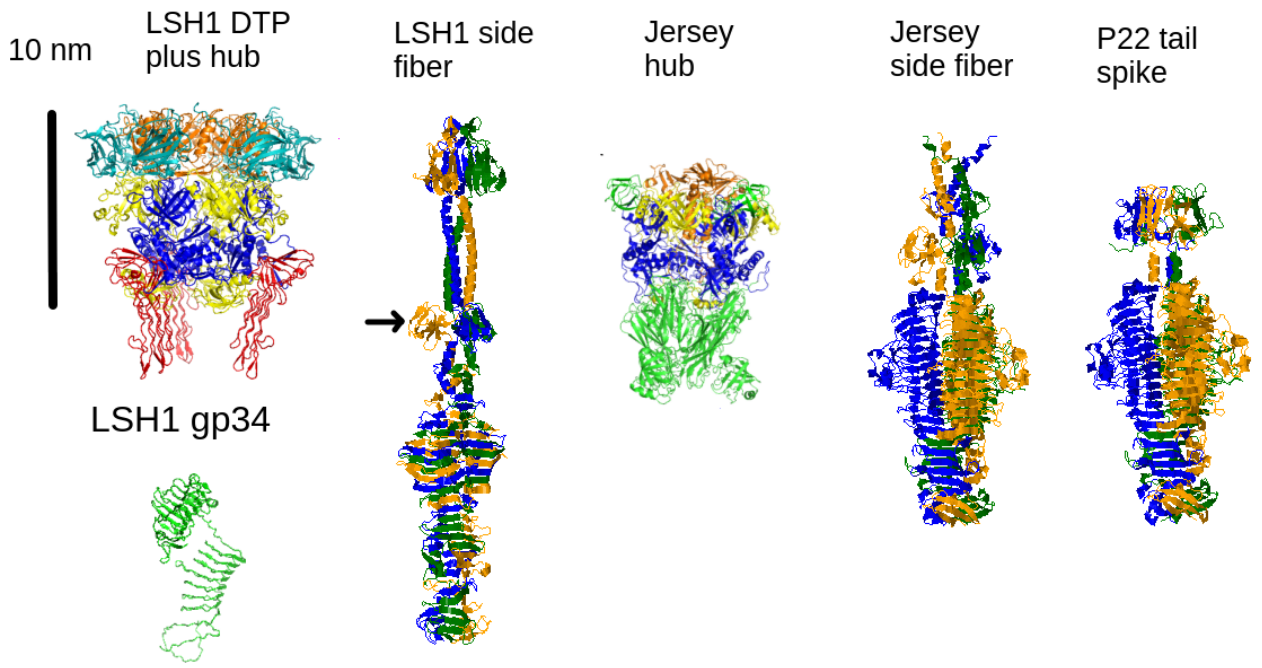

FIG. S10. Relative sizes of LSH1 and jersey hubs and associated components. The P22 tail spike was modeled by AlphaFold, but is comparable to PDB:8EAN. The LSH1 hub includes the distal tail protein (gp24) in orange and teal, and the tail hub protein in yellow, blue, and red (see main text). The Jersey hub is colored as in Fig. S7. The Jersey side fiber and P22 tail spike are colored by chain. The arrow by the LSH1 side fiber indicates a possible hinge region. LSH1 gp34 is included, although still poorly modeled, to give an indication of how much complexity it may possibly add.

REFERENCES

[1] Campbell PL, Duda RL, Nassur J, Conway JF, Huet A. Mobile Loops and Electrostatic Interactions Maintain the Flexible Tail Tube of Bacteriophage Lambda. J Mol Biol 2020;432:384-395. https://doi.org/10.1016/j.jmb.2019.10.031

[2] Zhao R, Du T, Ji Y, Ren Z, Jiang S, Ru H, Gu J. Characterization of the phage ΦK64 depolymerase S2-4 and its therapeutic effect against K1 serotype Klebsiella pneumoniae. Microbiol Res 2026;307:128475. https://doi.org/10.1016/j.micres.2026.128475

[3] Wang C, Duan J, Gu Z, Ge X, Zeng J, Wang J. Architecture of the bacteriophage lambda tail. Structure 2024;32:35–46.e3. https://doi.org/10.1016/j.str.2023.10.006

[4] Bryan AW Jr, Starner-Kreinbrink JL, Hosur R, Clark PL, Berger B. Structure-based prediction reveals capping motifs that inhibit β-helix aggregation. Proc Natl Acad Sci USA 2011;108:11099-104. https://doi.org/10.1073/pnas.1017504108

[5] Wang B, Dong S, Li FL, Ma XQ. Structural basis for the exolytic activity of polysaccharide lyase family 6 alginate lyase BcAlyPL6 from human gut microbe *Bacteroides clarus*. Biochem Biophys Res Commun 2021;547:111–17. https://doi.org/10.1016/j.bbrc.2021.02.040

[6] Luis AS, Briggs J, Zhang X, Farnell B, Ndeh D, Labourel A, Baslé A, Cartmell A, Terrapon N, Stott K, Lowe EC, McLean R, Shearer K, Schückel J, Venditto I, Ralet MC, Henrissat B, Martens EC, Mosimann SC, Abbott DW, Gilbert HJ. Dietary pectic glycans are degraded by coordinated enzyme pathways in human colonic *Bacteroides*. Nat Microbiol 2018;3:210-219. https://doi.org/10.1038/s41564-017-0079-1
